# Double-blind, placebo-controlled, proof-of-concept trial of bexarotene Xin moderate Alzheimer’s disease

**DOI:** 10.1186/s13195-016-0173-2

**Published:** 2016-01-29

**Authors:** Jeffrey L. Cummings, Kate Zhong, Jefferson W. Kinney, Chelcie Heaney, Joanne Moll-Tudla, Abhinay Joshi, Michael Pontecorvo, Michael Devous, Anne Tang, James Bena

**Affiliations:** Cleveland Clinic Lou Ruvo Center for Brain Health, 888 West Bonneville Avenue, Las Vegas, NV 89106 USA; Department of Psychology, University of Nevada, Las Vegas, NV USA; Avid Pharmaceuticals, Philadelphia, PA USA; Cleveland Clinic Quantitative Health Services, Cleveland, OH USA

**Keywords:** Clinical trial, Alzheimer’s disease, MRI, Amyloid, PET, Bexarotene, ApoE genotype

## Abstract

**Background:**

We assessed the impact of retinoid X receptor (RXR) agonist bexarotene on brain amyloid measured by amyloid imaging in patients with Alzheimer’s disease (AD) in a proof-of-concept trial.

**Methods:**

Twenty patients with AD [Mini Mental State Examination (MMSE) score 10–20 inclusive] with positive florbetapir scans were randomized to receive 300 mg of bexarotene or placebo for 4 weeks. The amyloid imaging result was the primary outcome. Whole-population analyses and prespecified analyses by genotype [apolipoprotein E ε4 (ApoE4) carriers and ApoE4 noncarriers] were conducted. Secondary outcomes included scores on the Alzheimer’s Disease Assessment Scale–Cognitive subscale, Alzheimer’s Disease Cooperative Study–Activities of Daily Living scale, MMSE, Clinical Dementia Rating scale, and Neuropsychiatric Inventory. Serum amyloid-β (Aβ) peptide sequences Aβ_1–40_ and Aβ_1–42_ measurements were collected as biomarker outcomes.

**Results:**

There was no change in the composite or regional amyloid burden when all patients were included in the analysis. ApoE4 noncarriers showed a significant reduction in brain amyloid on the composite measure in five of six regional measurements. No change in amyloid burden was observed in ApoE4 carriers. There was a significant association between increased serum Aβ_1–42_ and reductions in brain amyloid in ApoE4 noncarriers (not in carriers). There were significant elevations in serum triglycerides in bexarotene-treated patients. There was no consistent change in any clinical measure.

**Conclusions:**

The primary outcome of this trial was negative. The data suggest that bexarotene reduced brain amyloid and increased serum Aβ_1–42_ in ApoE4 noncarriers. Elevated triglycerides could represent a cardiovascular risk, and bexarotene should not be administered outside a research setting. RXR agonists warrant further investigations as AD therapies.

**Trial registration:**

ClinicalTrials.gov identifier NCT01782742. Registered 29 January 2013.

**Electronic supplementary material:**

The online version of this article (doi:10.1186/s13195-016-0173-2) contains supplementary material, which is available to authorized users.

## Background

There is an urgent need to develop new treatments for Alzheimer’s disease (AD). AD is currently the third leading cause of death in the United States [1] and costs the economy more annually than cardiovascular disease or cancer [2]. By 2050, the annual cost of AD to the U.S. economy will exceed $1 trillion [3]. All attempts to develop disease-modifying treatments for AD have failed [4].

Much current therapeutic research is focused on the amyloid hypothesis and means of redressing the imbalance between production and clearance of amyloid-β (Aβ) protein that leads to peptide aggregation, neurotoxicity, and formation of neuritic plaques [5]. Pharmaceutical approaches include reducing Aβ production, inhibiting its aggregation, and facilitating its removal [6].

The apolipoprotein E ε4 (*ApoE4*) allele is among the most potent risk factors for AD, increasing the risk of developing the disease and decreasing its age of onset [7]. ApoE4 has deleterious effects on protein metabolism, enhances Aβ_1–42_ aggregation, and impairs mitochondrial function [8]. The *ApoE4* genotype is associated with greater amyloid burden as measured by amyloid imaging [9]. *ApoE4* gene carriers have greater cortical and vascular amyloid deposition than noncarriers [10]. ApoE function and its ability to bind Aβ is influenced by its lipidation status, which is deficient in *ApoE4* carriers. Lipidation is determined by the ATP-binding cassette A1 (ABCA1), which is in turn under the control of nuclear retinoid X receptors (RXRs) [9, 11, 12]. RXR agonists induce the expression of ApoE and ABCA1 and increase ApoE lipidation, enhancing its ability to remove Aβ_1–42_ from the brain [13]. Cramer and colleagues [14] reported a marked effect of bexarotene, an RXR agonist, on Aβ levels in transgenic (Tg) mice. Eleven-month-old amyloid precursor protein-presenilin 1 mice treated with bexarotene for seven days had a 50 % reduction in plaque burden, significantly reduced levels of soluble and insoluble brain Aβ, and restoration of cognitive and memory functions. Attempts to confirm this observation have been only partially successful. Most follow-up studies reproduced effects on soluble Aβ; effects on amyloid plaques and behavioral outcomes were more variable [15–19].

Bexarotene is approved for treatment of cutaneous T-cell lymphoma and can be repurposed for treatment of other indications. It has been used off label for treatment of non–small cell lung cancer, breast cancer, and Kaposi’s sarcoma [20]. The observation that bexarotene may be effective in reducing the pathology and cognitive deficits of Tg animals with Aβ pathology suggested that assessment of bexarotene as a repurposed therapy for AD is warranted [21]. Off-label use of bexarotene in AD based on results in Tg animal systems is controversial because bexarotene is known to have substantial toxicity, commonly elevating triglyceride and cholesterol levels and increasing the risk of hypothyroidism [22–26]. One case report has suggested benefit from treatment of AD with bexarotene [27]. Rigorous evaluation of the potential benefits and harm of bexarotene in controlled trials is the optimal means of providing information on the therapeutic potential of this agent.

Drug development proceeds from preclinical observations in animal model systems to human studies, including proof-of-concept (POC), dose-finding, and large-scale pivotal phase III trials in preparation for regulatory submission. Clinical outcomes for trials of disease-modifying agents typically require large numbers of patients and long-term observations and do not lend themselves to POC investigations [28]. Although no biomarker has gained surrogate status and is known to predict clinical outcomes, biomarkers can be used to develop go–no-go decisions in early stages of drug development [29]. A drug lacking measurable biological effects would not be expected to have clinical benefits in larger trials and would not be advanced for further study. POC studies are exploratory, signal-seeking studies aimed at determining if a meaningful biological effect is present. Informative POC studies derisk drug development programs and increase the likelihood of success [30].

We conducted a biomarker-driven POC trial of bexarotene to determine if the provocative preclinical observations predict human biology and if studies with clinical outcomes should be pursued. We conducted a double-blind, randomized, placebo-controlled, parallel-group study of a single dose (300 mg/day) of bexarotene. We treated patients for 4 weeks in the double-blind portion of the study and continued observation for 4 additional weeks with all patients on treatment. The primary outcome was the effect of bexarotene on brain amyloid imaging after 1 month of treatment. We report the primary outcomes of the study.

## Methods

### Standard protocol, approvals, registrations, and patient consents

The study was approved by the Cleveland Clinic Institutional Review Board. Written informed consent was obtained from all patients (or guardians of patients) participating in the study. The trial is registered with ClinicalTrials.gov under the identifier NCT01782742.

### Trial design

The BExarotene Amyloid Treatment for Alzheimer’s Disease (BEAT AD) trial was a double-blind, placebo-controlled, parallel-group, single-site study with an allocation ratio in the 4 weeks of four bexarotene-treated patients to one placebo-treated patient (Additional File 1). In weeks 4–8, all patients received treatment with bexarotene. There were no changes in methodology after trial initiation.

### Participants

Patients were men women ages 50–90 years who met National Institute of Neurological Disorders and Stroke/Alzheimer’s Disease and Related Disorders Association criteria for AD dementia [31]. Each patient was required to have a positive florbetapir positron emission tomogram (PET) and a Mini Mental State Examination (MMSE) [32] score between 10 and 20 (inclusive) to be included in the trial. Positive amyloid PET was determined visually by two readers; if they disagreed, a third rater was invoked, and the majority reading was accepted. Subjects were required to have a study partner who could comply with all required study procedures. Subjects had at least 8 years of education, were capable of communicating effectively with the trial team, and had no uncontrolled medical illnesses. Treatment with any pharmacologic agent, including antidementia agents (cholinesterase inhibitors and/or memantine), had to be stable for at least 1 month before randomization. Patients were required to consent to ApoE genotyping. Patients were not stratified by ApoE genotype, and genotypes were not known until after unblinding of the trial.

### Interventions

Patients received bexarotene 75 mg or matching placebo twice daily for days 1–7. The dose was increased to 150 mg twice daily or matching placebo for days 8–28. Patients were treated with atorvastatin for elevated cholesterol levels and clofibrate for elevated triglyceride levels when these emerged in the course of the trial. These were not implemented until the end of week 4.

### Outcomes

The primary endpoint was the drug–placebo difference in change from baseline to week 4 in the composite Aβ burden of the brain as measured by standardized uptake value ratio (SUVr) with a florbetapir PET. A white matter standard for comparison was prespecified for the primary endpoint [33]. The effect of ApoE genotype was hypothesized to be influential, and change from baseline on treatment compared with placebo at week 4 on composite and regional Aβ burden according to *ApoE* genotype (*ApoE4* carriers compared with *ApoE4* noncarriers) was a declared primary outcome (Additional File 2). Determination of SUVr values using a cerebellar standard was included as a secondary outcome. Secondary clinical outcomes addressed change from baseline in the active treatment group compared with change from baseline in the placebo group at week 4. Changes in scores on the MMSE, Alzheimer’s Disease Assessment Scale–Cognitive subscale (ADAS-Cog) [34], Clinical Dementia Rating Sum of Boxes (CDR-SOB) [35], Neuropsychiatric Inventory (NPI) [36], and Alzheimer’s Disease Cooperative Study–Activities of Daily Living (ADCS-ADL) scale [37] were recorded. Change in Aβ_1–40_ and Aβ_1–42_ serum levels from baseline to week 4 were secondary biomarker outcomes. Safety and tolerability were measured by incidence of adverse events (AEs) or serious AEs, clinical laboratory data, vital signs, electrocardiograms, and magnetic resonance imaging (MRI) scans.

### Blinding and randomization

Patients were randomized in a 4:1 ratio of bexarotene to placebo as determined by a randomization sequence generated by Cleveland Clinic Department of Quantitative Health Sciences. Patients received either the active agent or identical appearing placebos. Patients were enrolled at the Cleveland Clinic Lou Ruvo Center for Brain Health, Las Vegas, NV, USA, and all study personnel, patients, and caregivers were blinded to allocation sequence and treatment status. Among trial personnel, only the Cleveland Clinic pharmacist had access to the randomization details.

### Sample size determination

There was no previous history of the use of bexarotene in AD to guide effect size estimates or standard deviations. The effects observed in Tg mice were dramatic and occurred within a short time frame. We hypothesized that a marked effect of the type seen in Tg animals could be observed in a sample size of 10 subjects with AD; thus, we planned recruitment of 20 subjects, with 4 assigned to placebo.

### ApoE genotyping

Whole blood was collected at baseline into PAXgene Blood DNA Tubes (PreAnalytiX, Hombrechtikon, Switzerland). DNA was extracted from the whole-blood samples using the PAXgene Blood DNA Kit according to kit specifications. DNA concentration and purity were assessed using a NanoDrop spectrophotometer (Thermo Scientific, Wilmington, DE, USA). Samples were genotyped for ApoE using the method and reported by Calero et al. [38].

### Serum amyloid analysis using enzyme-linked immunosorbent assays

Plasma was collected at baseline, 4 weeks, and 8 weeks. Plasma samples from each time point were analyzed using enzyme-linked immunosorbent assays for Aβ_1–40_ and Aβ_1–42_ (Wako Pure Chemical Industries, Osaka, Japan) according to kit instructions. All samples were run in triplicate. The samples were read on a SpectraMax Plus 384 spectrophotometer and plate reader (Molecular Devices, Sunnyvale, CA, USA).

### Amyloid imaging analysis

PET data were acquired from all 20 subjects after injection of 370 MBq (±10 %) of florbetapir at baseline and at week 4 for the primary analysis. PETs at all the sessions were acquired for 10 minutes at 50 minutes after injection. A Biograph mCT scanner (Siemens Healthcare, Malvern, PA, USA) was used for acquiring all PET data, and the images were reconstructed using three-dimensional ordered-subset expectation maximization to an image size of 128 × 128 pixels, slice thickness of 2 mm, and postreconstruction Gaussian filter of 2 mm. SUVr values at baseline were calculated using anatomically predefined, atlas-based cortical regions—medial orbital frontal, precuneus, parietal, temporal, anterior cingulate, and posterior cingulate [39]. SUVr values for week 4 and week 8 images were calculated using the prespecified primary method, implementing white matter as the internal standard [40] for measuring change (baseline to week 4).

### Statistical methods

Analyses were performed on the basis of the intention-to-treat principle (Additional File 2). Categorical factors were summarized using frequencies and percentages, while continuous measures were described using means and standard deviations. Comparisons of the changes between groups at 4 weeks were performed using linear models with change as the outcome. Results are presented as the mean and 95 % confidence interval. Spearman correlations were used to evaluate associations between changes in clinical measures. Each measure was evaluated for outliers. The small sample size limits the ability to detect departures from normality on which the results rely. Only very large differences between groups can be detected as significant, and larger samples would be needed to confirm any statistically significant findings. Analyses were performed using SAS software (version 9.3; SAS Institute, Cary, NC, USA) with a significance level of 0.05. In this exploratory POC trial, no adjustment for multiplicity of testing was performed.

## Results

### Participant flow and baseline features

**Fig. 1:**
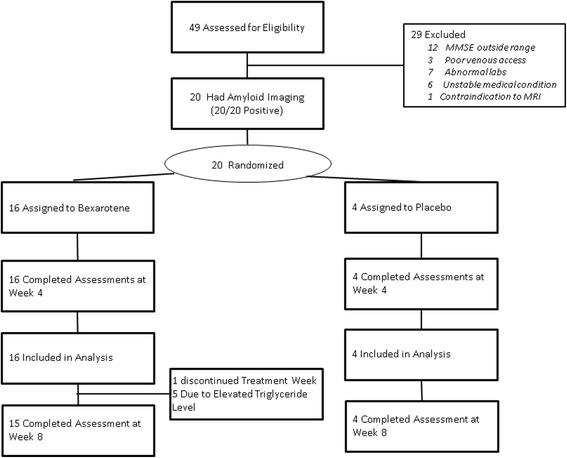
Disposition of subjects in the Bexarotene Amyloid Treatment for Alzheimer’s Disease trial. *MMSE* Mini Mental State Examination, *MRI* magnetic resonance imaging

Figure 1 shows the study disposition. Forty-nine subjects were screened, twenty-nine subjects did not meet the inclusion criteria and were not assessed further. Twenty patients had amyloid imaging and all twenty met the criteria for an elevated amyloid burden at baseline. One patient required three readers to reach a consensus on the presence of an abnormal amyloid signal. Twenty subjects were randomized, and all twenty completed the 4-week assessment. One subject was not continued into the weeks 4–8 open-label treatment period at the principal investigator’s request because of a greater than tenfold elevation in serum triglycerides.

### Study disposition

Patient recruitment began on 18 February 2013 and was completed on 5 June 2014. Recruitment was stopped when the prespecified number of subjects was reached, and the trial was completed 8 weeks after the last patient entered the trial.

Table 1 summarizes the baseline characteristics of the overall population and by treatment group. Patients were well matched for age; sex; years of education; and MMSE, ADAS-Cog, CDR-SOB, NPI, and ADCS-ADL scores. On average, the impairment of patients assigned to placebo was somewhat less severe than that of patients randomized to bexarotene. The recruited population included seven *ApoE4* noncarriers, seven *ApoE4* heterozygotes, and six ApoE4 homozygotes. Composite SUVr amyloid levels ranged from 1.37 to 1.41.

**Table 1:** Baseline characteristics, overall and by group

Characteristic	Total (*N* = 20)	Bexarotene (*n* = 16)	Placebo (*n* = 4)
Age at screening, yr	75.5 ± 6.8	74.9 ± 6.6	78.1 ± 8.0
Male sex	7 (35.0)	6 (37.5)	1 (25.0)
White race	19 (95.0)	15 (93.8)	4 (100.0)
Years of education	14.2 ± 4.7	14.7 ± 4.9	12.3 ± 3.3
Years of cognitive symptoms	4.3 ± 1.9	4.6 ± 1.9	2.8 ± 0.96
MMSE total score	14.4 ± 3.8	13.7 ± 3.7	17.0 ± 3.6
ADAS-Cog score^a^	48.0 ± 9.8	49.9 ± 8.9	40.3 ± 10.7
CDS-SOB score^a^	1.4 ± 0.56	1.4 ± 0.55	1.1 ± 0.63
NPI score^a^	8.4 ± 8.1	8.7 ± 8.6	7.0 ± 6.4
ADCS-ADL score^a^	55.9 ± 12.9	53.7 ± 13.1	64.5 ± 8.2
Composite SUVr	1.40 (0.11)	1.41 (0.09)	1.37 (0.16)
*ApoE4* genotype			
Noncarriers	7 (35.0)	4 (25.0)	3 (75.0)
Heterozygotes	7 (35.0)	6 (37.5)	1 (25.0)
Homozygotes	6 (30.0)	6 (37.5)	0 (0.0)

*ADAS-Cog* Alzheimer’s Disease Assessment Scale–Cognitive subscale, *ADCS-ADL* Alzheimer’s Disease Cooperative Study–Activities of Daily Living scale, *CDR-SOB* Clinical Dementia Rating Sum of Boxes, *MMSE* Mini Mental State Examination, *NPI* Neuropsychiatric Inventory, *SUVr* standardized uptake value ratio

Values presented as mean ± standard deviation, or count (%)

^a^ Data not available for all subjects; missing values: ADAS-Cog score = 1, CDS-SOB score = 3, NPI score = 4, NPI distress score = 4, ADCS-ADL score = 1

### Outcomes

The composite SUVr for all subjects showed no difference in change between the bexarotene-treated subjects and those receiving placebo (Table 2) following 4 weeks of treatment. The prespecified comparison of all subjects for specified brain regions also showed no drug–placebo difference.

**Table 2:** Comparisons of mean changes from baseline to 4 weeks in amyloid burden between treatment groups (white matter standard)

	Bexarotene		Placebo		Difference	
Factor	*N*	Mean (95 % CI)	*N*	Mean (95 % CI)	Mean (95 % CI)	*p* Value
All subjects						
Composite	16	−0.028 (−0.064, 0.008)	4	0.023 (−0.049, 0.096)	−0.052 (−0.133, 0.030)	0.22
Frontal medial orbital	16	−0.043 (−0.081, −0.006)	4	−0.021 (−0.096, 0.054)	−0.022 (−0.106, 0.062)	0.61
Anterior cingulate	16	−0.040 (−0.080, 0.000)	4	0.018 (−0.061, 0.098)	−0.059 (−0.147, 0.030)	0.20
Parietal	16	−0.003 (−0.034, 0.027)	4	0.044 (−0.018, 0.105)	−0.047 (−0.116, 0.022)	0.19
Posterior cingulate	16	−0.017 (−0.065, 0.031)	4	0.044 (−0.052, 0.141)	−0.061 (−0.169, 0.047)	0.27
Precuneus	16	−0.027 (−0.069, 0.014)	4	0.040 (−0.043, 0.122)	−0.067 (−0.159, 0.025)	0.16
Temporal	16	−0.038 (−0.075, 0.000)	4	0.016 (−0.059, 0.090)	−0.053 (−0.136, 0.030)	0.22
*ApoE4* noncarriers						
Composite	4	−0.097 (−0.155, −0.040)	3	0.047 (−0.019, 0.114)	−0.145 (−0.232, −0.057)	0.012
Frontal medial orbital	4	−0.076 (−0.146, −0.007)	3	0.005 (−0.075, 0.085)	−0.081 (−0.187, 0.025)	0.16
Anterior cingulate	4	−0.096 (−0.166, −0.026)	3	0.048 (−0.034, 0.129)	−0.143 (−0.251, −0.036)	0.029
Parietal	4	−0.068 (−0.107, −0.029)	3	0.065 (0.020, 0.110)	−0.133 (−0.193, −0.073)	0.002
Posterior cingulate	4	−0.113 (−0.180, −0.046)	3	0.074 (−0.004, 0.151)	−0.187 (−0.289, −0.084)	0.007
Precuneus	4	−0.127 (−0.188, −0.066)	3	0.062 (−0.008, 0.132)	−0.189 (−0.282, −0.096)	0.004
Temporal	4	−0.104 (−0.162, −0.045)	3	0.031 (−0.037, 0.098)	−0.134 (−0.224, −0.045)	0.018
*ApoE4* carriers						
Composite	12	−0.005 (−0.041, 0.031)	1	−0.048 (NA)	NA	NA
Frontal medial orbital	12	−0.033 (−0.074, 0.009)	1	−0.099 (NA)	NA	NA
Anterior cingulate	12	−0.022 (−0.062, 0.019)	1	−0.069 (NA)	NA	NA
Parietal	12	0.018 (−0.013, 0.050)	1	−0.019 (NA)	NA	NA
Posterior cingulate	12	0.015 (−0.035, 0.065)	1	−0.044 (NA)	NA	NA
Precuneus	12	0.006 (−0.031, 0.043)	1	−0.027 (NA)	NA	NA
Temporal	12	−0.015 (−0.055, 0.024)	1	−0.030 (NA)	NA	NA

*ApoE4* apolipoprotein E ε4, *CI* confidence interval, *NA* not applicable

**Fig. 2:**
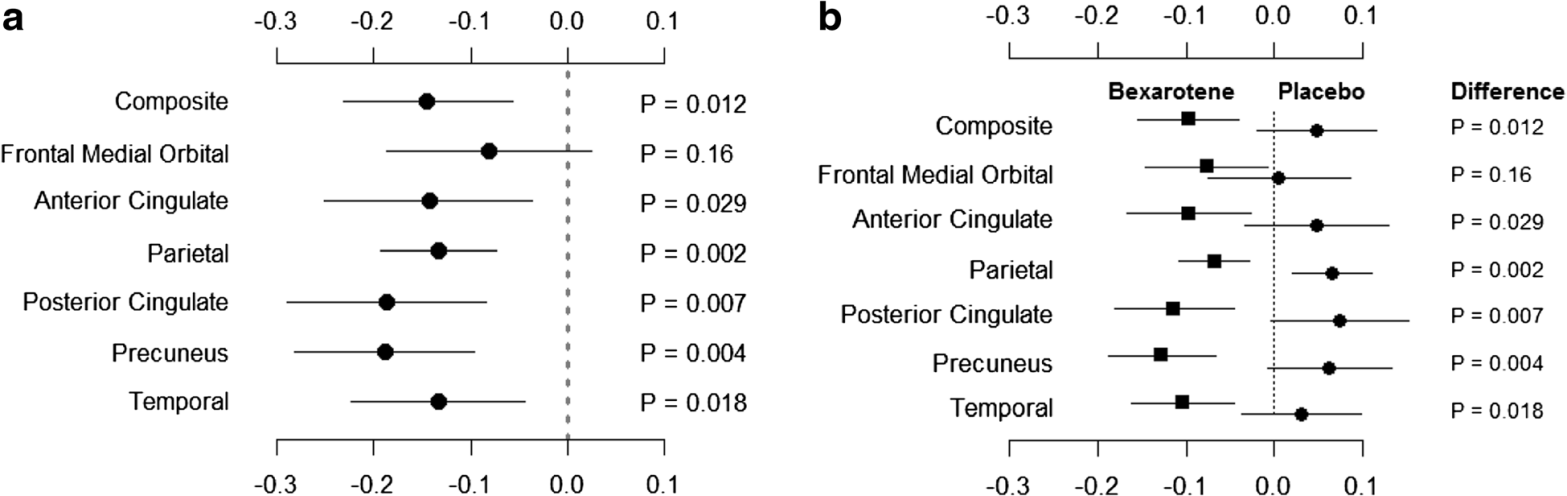
Panel **a** shows the difference in mean amyloid burden and 95 % confidence intervals for changes from baseline at 4 weeks between bexarotene and placebo groups. Panel **b** shows mean group changes and 95 % confidence intervals at 4 weeks. P-values reflect the comparisons of mean change between groups

The prespecified analysis of drug–placebo difference in change from baseline of amyloid burden by ApoE genotype demonstrated a significant reduction of Aβ on the composite measure and a reduction of Aβ in anterior cingulate cortex, parietal cortex, posterior cingulate cortex, precuneus, and temporal cortex in *ApoE4* noncarriers using the declared white matter standard (Table 2; Figure 2). There was no significant difference in change from baseline to 4 weeks in amyloid measures among *ApoE4* carriers receiving bexarotene (heterozygotes, homozygotes, or combined) compared with those on placebo. Analysis of changes at week 4 for composite and regional changes in Aβ using a cerebellar comparison standard showed no differences between drug and placebo for all subjects, including *ApoE4* noncarriers (data not shown).

Analysis of MMSE scores showed a significant difference between change from baseline with bexarotene compared with placebo in favor of placebo in ApoE4 noncarriers (*p* = 0.026) at week 4. This was due to an improvement in MMSE scores in the placebo group. There was no drug-placebo difference between the drug and placebo groups, considered together or by genotype on ADAS-Cog, NPI, and ADCS-ADL (Table 3).

**Table 3:** Comparisons of changes from baseline to 4 weeks in clinical measures between treatment groups

	Bexarotene		Placebo		Difference	
Factor	Number of subjects	Mean (95 % CI)	Number of subjects	Mean (95 % CI)	Mean (95 % CI)	*p* Value
All subjects						
MMSE	16	0.75 (−0.78, 2.28)	4	1.75 (−1.32, 4.82)	−1.00 (−4.43, 2.43)	0.57
ADAS-Cog	16	0.38 (−2.15, 2.90)	4	−0.25 (−5.31, 4.81)	0.63 (−5.03, 6.28)	0.83
CDR-SOB	16	0.00 (0.00, 0.00)	4	0.00 (0.00, 0.00)	0.00 (0.00, 0.00)	NA^a^
NPI	16	−2.63 (−6.78, 1.53)	4	−2.25 (−10.57, 6.07)	−0.38 (−9.67, 8.92)	0.94
ADCS-ADL	16	−1.94 (−4.86, 0.99)	4	−6.50 (−12.35, −0.65)	4.56 (−1.98, 11.10)	0.18
*ApoE4* noncarriers						
MMSE	4	−0.25 (−2.12, 1.62)	3	3.67 (1.51, 5.82)	−3.92 (−6.77, −1.06)	0.026
ADAS-Cog	4	−3.00 (−7.04, 1.04)	3	−0.33 (−4.99, 4.33)	−2.67 (−8.83, 3.50)	0.41
CDR-SOB	4	0.00 (0.00, 0.00)	3	0.00 (0.00, 0.00)	0.00 (0.00, 0.00)	NA^a^
NPI	4	−1.25 (−12.43, 9.93)	3	−3.33 (−16.25, 9.58)	2.08 (−15.00, 19.16)	0.81
ADCS-ADL	4	−4.75 (−10.69, 1.19)	3	−7.67 (−14.53, −0.81)	2.92 (−6.16, 11.99)	0.53

*ADAS-Cog* Alzheimer’s Disease Assessment Scale–Cognitive subscale, *ADCS-ADL* Alzheimer’s Disease Cooperative Study–Activities of Daily Living scale, *ApoE4* apolipoprotein E ε4, *CDR-SOB* Clinical Dementia Rating Sum of Boxes, *CI* confidence interval, *MMSE* Mini Mental State Examination, *NPI* Neuropsychiatric Inventory

^a^Not applicable (NA); *p* value was not calculated, because no change was observed in any patient at 4 weeks.

A significant increase in serum Aβ_1–42_ was seen at week 4 in the bexarotene group compared with those on placebo when all subjects were included. Increases in serum Aβ_1–42_ correlated with decreased cortical amyloid in treated *ApoE4* noncarriers (Table 4), not in treated *ApoE4* carriers (data not shown). There were no correlations between Aβ_1–40_ serum level changes and cortical amyloid changes.

**Table 4:** Spearman correlations between changes from baseline to 4 weeks (*ApoE4* noncarriers)

Measure	Brain region	*r* Value	95 % CI	*p* Value
Cholesterol change	Composite SUVr	−0.66	(−1.00, 0.39)	0.16
	Frontal medial orbital	−0.54	(−1.00, 0.62)	0.27
	Anterior cingulate	−0.66	(−1.00, 0.39)	0.16
	Parietal	−0.89	(−1.00, −0.24)	0.019
	Posterior cingulate	−0.66	(−1.00, 0.39)	0.16
	Precuneus	−0.66	(−1.00, 0.39)	0.16
	Temporal	−0.66	(−1.00, 0.39)	0.16
Triglycerides change	Composite SUVr	−0.89	(−1.00, −0.24)	0.019
	Frontal medial orbital	−0.77	(−1.00, 0.11)	0.072
	Anterior cingulate	−0.83	(−1.00, −0.05)	0.042
	Parietal	−1.00	(−1.00, −1.00)	
	Posterior cingulate	−0.89	(−1.00, −0.24)	0.019
	Precuneus	−0.89	(−1.00, −0.24)	0.019
	Temporal	−0.89	(−1.00, −0.24)	0.019
Aβ_1–42_ change	Composite SUVr	−0.83	(−1.00, −0.05)	0.042
	Frontal medial orbital	−0.71	(−1.00, 0.26)	0.11
	Anterior cingulate	−0.94	(−1.00, −0.48)	0.005
	Parietal	−0.54	(−1.00, 0.62)	0.27
	Posterior cingulate	−0.83	(−1.00, −0.05)	0.042
	Precuneus	−0.83	(−1.00, −0.05)	0.042
	Temporal	−0.83	(−1.00, −0.05)	0.042
Aβ_1–40_ change	Composite SUVr	−0.37	(−1.00, 0.92)	0.47
	Frontal medial orbital	−0.31	(−1.00, 1.00)	0.54
	Anterior cingulate	−0.26	(−1.00, 1.00)	0.62
	Parietal	0.20	(−1.00, 1.00)	0.70
	Posterior cingulate	−0.37	(−1.00, 0.92)	0.47
	Precuneus	−0.37	(−1.00, 0.92)	0.47
	Temporal	−0.37	(−1.00, 0.92)	0.47

*CI* confidence interval, *SUVr* standardized uptake value ratio

Correlational analyses showed significant correlations between elevations in cholesterol and reduced amyloid levels in one cortical region (parietal) and between elevations of triglycerides and reduced cortical amyloid in four of the six cortical regions and in the composite cortical amyloid measure in ApoE4 noncarriers (Table 4). There were no significant correlations between serum cholesterol and triglyceride levels and cortical amyloid change measures in *ApoE4* carriers.

### Safety

Fifteen of twenty subjects had increases in triglyceride levels to greater than 200 mg/dl. Eleven subjects had increases in cholesterol levels to greater than 300 mg/dl. Other AEs included delusions, dizziness, toe blister, dry cough, and diverticulitis (one each). No amyloid-related imaging abnormalities of either the effusion or hemorrhagic type were seen on MRI scans at week 4 or week 8.

## Discussion

This POC trial had a small number of participants, and ApoE genotype subgroups were also small, limiting the conclusions that can be drawn. Patients were excluded if they had abnormal lipid levels at baseline (six subjects were excluded on this basis) or if they had any medical illness that was not adequately controlled (six were excluded on this basis). We do not know the effects of bexarotene in patients who were in excluded categories, and the results should be generalized with caution. SUVr may be susceptible to flow effects, and any impact of bexarotene on blood flow could affect interpretation of the results. Bexarotene is not known to alter blood flow. We chose a brief exposure period in this POC study to explore potential acute effects of bexarotene. Longer exposures are necessary to assess effects on clinical outcomes and to determine if removal of amyloid in *ApoE4* carriers is observed with longer exposures.

Subjects recruited into this study were typical of patients with moderate AD with MMSE scores in the range of 10–20. They received the usual standard of care with regard to antidementia therapies. All patients were determined by amyloid imaging to have an elevated brain amyloid burden at baseline meeting clinical and biomarker data for AD dementia [41].

The primary outcome of this study for all subjects was negative; no difference was seen between treatment with bexarotene and placebo when all patients were included in the analysis. Using the prespecified ApoE genotype-based analyses, we observed a highly significant reduction in brain amyloid on the composite measure and in five of six cortical regions of interest among *ApoE4* noncarriers. The *ApoE4* noncarriers had reductions in amyloid burden in all measured cortical regions after 4 weeks of treatment with bexarotene, and those on placebo had slight increases in burden (Table 2; Figure 2)

.

There was a significant correlation between reduced cortical amyloid and elevated serum levels of Aβ_1–42_, suggesting that soluble forms of amyloid were transferred from brain to blood, although a direct effect of bexarotene on serum Aβ_1–42_ binding has not been excluded. In *ApoE4* noncarriers, there were significant correlations between elevated serum triglyceride levels and decreased cortical amyloid. Bexarotene is an RXR/liver X receptor agonist; the latter mechanism is linked to elevated triglyceride levels.

Reductions in brain amyloid and correlations of reduced brain amyloid with serum Aβ_1–42_ were seen only in *ApoE4* noncarriers. In Tg mice, ApoE4 presence results in more compact plaques; ApoE3 and ApoE2 mice have more diffuse plaques, which may result in more ready mobilization of amyloid with therapy [42]. ApoE4 has a deficient lipidation status, and increased lipidation by bexarotene may be specific to noncarriers [43]. Alternatively, the apparently genotype-specific effects of bexarotene may relate to a potential different time course of response in *ApoE4* carriers and noncarriers.

There were no significant changes in cognitive measures. The treatment was brief, the period with relatively decreased amyloid was correspondingly brief, and the trial duration may not have been long enough to observe cognitive benefit. The trial was not powered to observe cognitive change.

Bexarotene has effects on serum lipids that may increase the risk of stroke and heart attack, and this agent should not be used off label for clinical treatment until cognitive efficacy is demonstrated in a clinical trial.

## Conclusions

The results of the BEAT AD trial suggest that bexarotene may lower brain amyloid levels in patients with mild to moderate AD who do not carry the *ApoE4* gene. This biomarker-based POC trial supports a mechanism-based biological effect of bexarotene on targets relevant to AD pathogenesis. The potential clinical and biological effects of RXR therapeutics in AD and in biologically derived subtypes of AD warrant further investigation.

## Additional files

Additional file 1:
**BexProtocol-Ver#2-8-1-12.** (DOC 299 kb) Additional file 2:
**BEATADStatisticalPlanFinal_1 3_20141209.** (DOC 467 kb)

## Abbreviations

ABCA1: ATP-binding cassette A1
AD: Alzheimer’s disease
ADAS-Cog: Alzheimer’s Disease Assessment Scale–Cognitive subscale
ADCS-ADL: Alzheimer’s Disease Cooperative Study–Activities of Daily Living scale
AE: adverse event
ApoE4: apolipoprotein E ε4
ATP: adenosine triphosphate
Aβ: amyloid-β
BEAT AD trial: Bexarotene Amyloid Treatment for Alzheimer’s Disease trial
CDR-SOB: Clinical Dementia Rating Sum of Boxes
CI: confidence interval
MMSE: Mini Mental State Examination
MRI: magnetic resonance imaging
NPI: Neuropsychiatric Inventory
PET: positron emission tomography
POC: proof of concept
RXR: retinoid X receptor
SUVr: standardized uptake value ratio
Tg: transgenic
