# Supplementary material for: Double-blind, placebo-controlled, proof-of-concept trial of bexarotene Xin moderate Alzheimer’s disease
Source: Alzheimers Res Ther. 2016 Jan 29;8:4. doi: 10.1186/s13195-016-0173-2 (PMC4731943; doi:10.1186/s13195-016-0173-2)
Supplement: Additional file 1: — BexProtocol-Ver#2-8-1-12. (DOC 299 kb) [file 13195_2016_173_MOESM1_ESM.doc]

Center for Clinical Research

# NEUROLOGICAL INSTITUTE OF CLEVELAND CLINIC

# THE LOU RUVO CENTER FOR BRAIN HEALTH

PHASE II STUDY EVALUATING THE SAFETY
AND BIOMARKER EFFICACY OF BEXAROTENE IN PATIENTS
WITH MILD TO MODERATE ALZHEIMER’S DISEASE:

BExarotene Amyloid Treatment for Alzheimer’s Disease (BEAT AD)

Sponsor Investigator: Jeffrey L. Cummings, MD, ScD

Director, Cleveland Clinic Lou Ruvo Center for Brain Health

888 West Bonneville Avenue

Las Vegas, NV 89106

702-483-6031 (phone)

702-483-6028 (fax)
cumminj@ccf.org

Co-Investigators:

Dr. Charles Bernick bernicc@ccf.org

Dr. Gabriel Léger legerg@ccf.org

ABBREVIATIONS

*AA Alzheimer’s Association*

*ABETA (Aß) Amyloid Beta Protein*

*AD Alzheimer’ Disease*

*****ADAS-cog Alzheimer's Disease Assessment Scale Cognitive Portion*****

*ADCS-ADL Alzheimer’s Disease Cooperative Study Activities of Daily Living Scale*

*AE Adverse Event*

*ApoE Apolipoprotein E*

*APP Amyloid precursor protein*

*ARIA-E Amyloid-related imaging abnormalities – effusion*

*BBB Blood brain barrier*

*CA Composite Assessment of Index Lesion Disease Severity*

*CBC Complete Blood Count*

*CCF Cleveland Clinic Foundation*

*CDRSOB Clinical Dementia Rating Sum of Boxes*

*CRF Case Report Form*

*CSF Cerebrospinal fluid*

*CTCL Cutaneous T-cell lymphoma*

*FDA Food and Drug Administration*

*GCP Good Clinical Practice*

*HIPPA Health Insurance Portability and Accountability Act of 1996*

*ICH International Conference of Harmonization (ICH)*

*IRB Institutional Review Board*

*ITT Intent to Treat*

*IU International Units*

*IWG International Work Group*

*LOCF Last observation carried forward*

*MMSE Mini-Mental State Examination*

*MRI Magnetic resonance imaging*

*NIA National Institute on Aging*

*NINCDS/ADRDA National Institute of Neurologic and Communicative Disorders and Stroke-Alzheimer’s Disease and Related Disorders Association*

*NMDA N-methyl-D-aspartate*

*****NPI***** *Neuropsychiatric Inventory*

*PET Positron Emission Tomography*

*PHI Protected Health Information*

*PIB Pittsburgh Compound B*

*QHS Quantitative Health Sciences*

*SAE Serious Adverse Event*

*sPPP-α soluble amyloid precursor protein- alpha*

*sAPP-β soluble amyloid precursor protein-beta*

*SUVR Standard Uptake Value Regional*

*Tg Transgenic*

TABLE OF CONTENTS

Title Page 1

Abbreviations 2

Table of Contents 3

Research Schema 6

1.0 Introduction 7

1.1 Background 7

1.2 Investigational Agent 9

1.3 Preclinical Data 10

1.4 Clinical Data to Date 10

1.5 Dose Rationale and Risk/Benefits 11

2.0 Study Objectives 12

3.0 Study Design 13

3.1 General Design 13

3.2 Primary Study Endpoints 13

3.3 Secondary Study Endpoints 13

3.4 Primary Safety Endpoints 13

4.0 Subject Selection and Withdrawal 14

4.1 Inclusion Criteria 14

4.2 Exclusion Criteria 14

4.3 Subject Recruitment and Screening 15

4.4 Early Withdrawal of Subjects 15

4.5 Data Collection and Follow-up for Withdrawn Subjects 15

5.0 Study Drug 16

5.1 Description 16 13

5.2 Treatment Regimen 16

5.3 Method for Assigning Subjects to Treatment Regimen 16

5.4 Preparation and Administration of Study Treatment 16

5.5 Subject Compliance Monitoring 16

5.6 Prior and Concomitant Therapy 17

5.7 Packaging 17

5.8 Blinding of Study Drug 17

5.9 Receiving, Storage, Dispensing and Return 17

5.9.1 Receipt of Drug Supplies 17

5.9.2 Storage 17

5.9.3 Dispensing of Study Drug 17

5.9.4 Return or Destruction of Study Drug 18

6.0 Study Procedures 18

6.1 Pre-registration and Screening 18

6.2 Visit 2 18

6.3 Visit 3 19

6.4 Visit 4 19

6.5 Visit 5 19

6.6 Visit 6 19

6.7 Visit 7 19

6.8 Unscheduled visit 20

6.9 Study Calendar of Procedures 20

6.10 Laboratory Procedures 20

7.0 Statistical Plan 21

7.1 Sample Size Determination 21

7.2 Statistical Methods 21

7.3 Subject Population(s) for Analysis 21

7.4 Amyloid Imaging Analysis 22

8.0 Safety and Adverse Events 22

8.1 Definitions 22

8.2 Recording of Adverse Events 22

8.3 Reporting of Serious Adverse Events 23

8.3.1 Study Sponsor Notification by Investigator 23

8.3.2 IRB Notification by Investigator 23

8.3.3 FDA Notification by Sponsor-Investigator 24

8.4 Unblinding Procedures 24

8.5 Stopping Rules 24

8.6 Medical Monitoring 24

8.6.1 Internal Data Safety Monitoring Board (DSMB) 24

9.0 Data Handling and Record Keeping 24

9.1 Confidentiality and Privacy 24

9.2 Source Documents 25

9.3 Case Report Forms 25

9.4 Records Retention 25

9.5 Database 25

10.0 Study Monitoring, Auditing, and Inspecting 25

10.1 Study Monitoring Plan 25

10.2 Auditing and Inspecting 25

11.0 Ethical Considerations 25

12.0 Study Finances 26

12.1 Funding Source 26

12.2 Conflict of Interest 26

12.3 Subject Stipends or Payments 26

13.0 Publication Plan 26

14.0 References 26

15.0 Attachments 28

RESEARCH SCHEMA

Phase II clinical study of bexarotene

1 2 3 4 6 7

1 – screening visit, MRI, amyloid imaging, slit lamp examination

2 – baseline (within 45 days of screening)

3 – week 1; increase dose from 1 BID to 2 BID

4 – week 2; blood draws, safety visit

5 – week 4; blood draws, slit lamp examination, MRI, amyloid imaging, end of double blind period; initiation of open label extension

6 – week 8; blood draws, slit lamp examination, amyloid imaging, MRI, amyloid imaging, end of

open label extension

7 – week 10; end of study

PHASE II STUDY EVALUATING THE SAFETY AND CLINICAL
AND BIOMARKER EFFICACY OF BEXAROTENE IN PATIENTS WITH
MILD TO MODERATE ALZHEIMER’S DISEASE:

BExarotene Amyloid Treatment for Alzheimer’s Disease (BEAT AD)

1.0 INTRODUCTION

- 1. Background

Alzheimer’s disease (AD) is rapidly growing in frequency as the population of the U.S. and the world ages. The current 5.5 million victims is projected to grow to 13 million by 2050 if no means of ameliorating this disease is found. Costs of the care of AD patients are sky-rocketing and it is currently anticipated that annual costs by the year 2050 will approach one trillion dollars. An individual develops dementia of the Alzheimer type every 70 seconds in the United States. The global burden of the disease will reach an estimated 100 million victims by the year 2050 (Thies et al, 2011; Alzheimer’s Association 2012).

Alzheimer’s disease has traditionally been identified as a dementia syndrome (McKhann et al, 1984). It is now recognized that AD occurs along a spectrum of severity from having no symptoms but with biomarkers indicative of the presence of AD (preclinical AD or at risk for AD), to a state with symptoms but no functional impairment (mild cognitive impairment [MCI] due to AD or prodromal AD), to AD dementia (Dubois et al, 2007, 2010; Sperling et al, 2011; Albert et al, 2011; McKhann et al, 2011). The criteria for predemetnia AD have been advanced by the International Work Group (IWG)(Dubois et al, 2007. 2010) and the National Institute (NIA)/Alzheimer’s Association (AA)(Sperling et al, 2011; Albert et al, 2011; McKhannn eet al, 2011). Amyloid abnormalities --- low cerebrospinal fluid (CSF) amyloid beta protein (Aβ) and positive amyloid imaging --- are characteristic of all phases of AD from the asymptomatic onset to the advanced phases of the disease (Rowe and Villemagne, 2011). AD includes an autosomal dominant form with mutations of amyloid precursor protein, presenilin 1 or presenilin 2 and idiopathic late onset forms. The late onset forms may occur with or without AD risk genes such as ApoE e4.

When these predementia forms of AD are considered, the total number of victims in the US and globally is much larger than stated above. The demographic data are based on the prevalence of AD dementia and including preclinical or prodromal forms of AD will greatly increase the number of individuals affected with this disorder.

There is an urgent need to find new therapies that will delay the onset, slow the decline or improve the symptoms of AD.

AD is linked to the aggregation and neurotoxicity of abnormal proteins in the brain. Two proteins have been identified to have principle roles in the progression of AD; Aß and tau protein (Ittner and Gotz, 2011). Aß appears to be the first protein to accumulate in the brain of the AD patients and is identifiable as resident in the brain for up to a decade before the onset of symptoms. Whether Aß continues to play a role in the ongoing disease process is controversial, but the spread of Aß pathology, the increase in the number of plaques in cerebral cortex, and the linkage between Aß and tau all suggest that Aß accumulation and aggregation continues throughout the disease course. Tau cell-to-cell transmission, aggregation, and hyper-phosphorylation are linked to cell dysfunction and cell death. Measures of cognitive decline and cerebral atrophy are more closely linked to tau than to Aß pathology.

Agents addressing that pathologic cascade of AD are currently being investigated to determine their potential therapeutic benefit in AD (Lukiw, 2012). Approved medications result in improved cholinergic function (cholinesterase inhibitors) or reduced calcium-related cell injury mediated via N-Methyl-D-Aspartate (NMDA) receptors (memantine)(Massoud and Leger, 2011). Many agents currently under development are aimed at disease modification through effects on Aß, tau, mitochondrial function, neuro -protection, apoptosis, or neuro-regeneration (Huang and Mucke, 2012). A variety of approaches to Aß pathology are under investigation, including beta secretase inhibition, gamma-secretase inhibition, alpha-secretase enhancement, Aß degradation facilitation, inhibition of aggregation, reduction of amyloid precursor protein (APP), Aβ export across the blood brain barrier (BBB), and Aß removal through microglial activity or peripheral sink induction (Lukiw, 2012). Two general forms of Aβ are recognized – soluble and insoluble – with the greatest neurotoxicity attributed to the soluble form consisting of Aβ oligomers. The oligomers fibrillize to form insoluble amyloid characteristic of plaques. Plaques may serve as a reservoir for soluble amyloid.

Amyloid imaging identifies amyloid plaques composed of insoluble fibrillar amyloid in the brain (Rowe and Villemagne, 2011), and reduction in the amyloid plaque burden of the brain can be documented with amyloid imaging. Microglial activation in AD can also be documented with specialized imaging techniques (Yokokura et al, 2011).

Retinoid X receptors (RXR) are nuclear receptors that have been linked to numerous metabolic pathways relevant to AD and to Aβ production and removal (Liang et al, 2004; Goodman et al, 2006; Tippmann et al, 2009; Suon et al, 2010; Jarvis et al, 2010; Cramer et al, 2012). RXRs up-regulate alpha-secretase via ADAM10 and decrease production of Aβ (Tippmann et al, 2009). RXR signaling has been shown to antagonize both intracellular and extracellular Aβ production and to prevent Aβ-related cell death (Jarvis et al, 2010). RXRs mediate apolipoprotein E (Apo E) expression that has been linked to the removal of Aβ from the brain (Liang et al, 2004; Suon et al, 2010). RXRs also increase insulin sensitivity (Mukherjee et al, 1997); reduced insulin signaling is present in AD and insulin sensitizing agents are being assessed for their potential therapeutic role in AD (rosiglitazone, pioglitazone).

RXR agonists are possible therapies for AD dementia and also for other forms of predementia AD such as preclinical and prodromal AD or MCI due to AD. These states are defined by amyloid imaging and CSF Aβ abnormalities, and in the case of prodromal AD/MCI due to AD by the presence of cognitive impairment without functional compromise. Moreover, ApoE may be able to bind to other proteins and RXR agonists may have beneficial effects in other neurodegenerative disorders linked to protein aggregation including Parkinson’s disease, dementia with Lewy bodies, frontotemporal lobar degeneration, frontotemporal dementia, Pick’s disease, progressive supranuclear palsy, corticobasal degeneration, multisystem atrophy and muscle and nerve diseases with protein aggregation.

The anti-cancer agent bexarotene is an RXR agonist that reduces Aß in the brain in experimental models of AD (Cramer et al, 2012). It increases expression of ApoE and activates microglia. Bexarotene reduced brain Aß in transgenic (tg) mice with mutations that cause Aβ deposition and it improved cognitive performance in animals with increased brain Aβ.

The current Phase II protocol is designed to investigate the efficacy of bexarotene in patients with mild to moderate AD dementia. Safety is a key outcome and the principal measure of efficacy is change in Aß burden on amyloid imaging. A key secondary outcome is cognitive change on the ****Alzheimer's Disease Assessment Scale (ADAS-cog) (Rosen et al, 1984)****, and other secondary outcomes include change on the Clinical Dementia Rating sum of boxes (CDRSOB) (Hughes et al, 1982; Lynch et al, 2006; Morris et al, 1997; Burke et al, 1988), the Neuropsychiatric Inventory (NPI) (Cumnings et al, 1994), and the Alzheimer’s Disease Cooperative Study Activities of Daily Living Scale (ADCS-ADL) (Galasko et al, 1997).

The amyloid imaging agent to be used in the study is florbetapir (av-345; Amyvid™), an Aβ ligand used with positron emission tomography (PET) to establish the presence of fibrillary amyloid plaques (Clark et al, 2011). It has recently been approved by the Food and Drug Administration (FDA) and is not an investigational agent. Amyvid is not approved or indicated as an outcome measure for clinical trials and use of the agent in this trial will be under the auspices of the Investigational New Drug (IND) application held by Eli Lilly Pharmaceuticals (NDA 202, 008; with permission).

- 1. Investigational Agent

1.2.a Bexarotene

Bexarotene is a member of a sub-class of retinoids that selectively activate RXRs. RXRs increase the expression of ApoE that enhances Aß degradation through proteolysis. Agonists of RXRs also act on macrophages and microglia to stimulate their conversion to activated states and promote phagocytosis. Aβ is a protein deposited in the brain of AD patients and thought to be responsible for initiating the cascade of events leading to memory impairment, cell death, and eventual death. Aβ can be removed by microglia and activation of microglia by RXRs is a potential mechanism for stimulating removal of Aβ from the brain. Increased processing of Aß through upregulation of ApoE-and removal of lapidated Aβ is another action of bexarotene that may be active in amyloid removal from the brain.

Bexarotene is approved by the US Food and Drug Administration (FDA) for the treatment of cutaneous T-cell lymphoma (CTCL) in patients who are refractory to at least one prior systemic therapy (Tegretin™ Package Insert).

The urgent need for new treatments of AD in conjunction with the many observations regarding the utility of RXRs in mechanisms relevant to AD combined with the fact that bexarotene is an approved agent with known pharmacology and toxicity suggests that bexarotene should be repurposed and tested in patients with AD. Patients must be properly informed of the absence of human data supporting the use of this agent in AD and the possible side effects associated with taking bexarotene.

Bexarotene has a T_max_ of approximately two hours and a terminal half-life of approximately seven hours. Both area under the curve and C_max_ are elevated after a fat-containing meal. Bexarotene is 99% protein bound and is metabolized into four metabolites that are active in in-vitro assays of RXRs. Bexarotene is eliminated primarily through the hepatobiliary system with less than 1% excreted unchanged in the urine. Bexarotene is metabolized by the cytochrome P4503A4 enzyme system. Therefore, CYP3A4 inhibitors (including grapefruit juice) would be expected to lead to increased plasma bexarotene levels. Bexarotene causes malformations when administered to pregnant rats and should not be administrated to pregnant women (Tegretin™ Package Insert)..

Bexarotene is associated with a variety of side-effects. About 70% of patients with CTCL treated with the agent had fasting triglyceride levels greater than 2.5 times the upper limit of normal. Similarly, cholesterol elevations above 300mg/dL occurred in approximately 60% to 75% of patients. Antilipemic therapy mitigates this response. Fasting blood lipid determination should be performed before bexarotene therapy and should be monitored during therapy. Acute pancreatitis was seen in 4 of 152 cancer patients. Elevation in serum liver functions was observed in 5% (SGOT), 2% (SGPT), and 0% (bilirubin) of exposed patients. Biochemical or clinical hypothyroidism occurs in approximately half of patients treated; 18% of patients have reversible leukopenia in the range of 1000 to <3000 WBC/mm^3^. Posterior subcapsular cataracts were observed in pre-clinical toxicity studies in rats and dogs. 15 of 79 patients who had serial slit lamp examinations were shown to have new cataracts or worsening of previous cataracts. Because of the relationship of bexarotene to vitamin A, patients will be advised to limit vitamin A supplements to less than 15,000 IU/day to avoid potential additive toxic effects. Retinoids as a class have been associated with photosensitivity and excessive sun exposure should be avoided. Bexarotene is not mutagenic in bacteria or mammalian cells but has been associated with testicular degeneration in dogs. Bexarotene is teratogenic in pregnant rats (Tegretin™ Package Insert and Investigator’s Brochure).

There is a substantial experience with bexarotene in older patients. In clinical studies of CTCL, 64% of patients were 60 years old or older and 33% were 70 years old or older. There were no overall differences in safety between patients younger and older than 70 years.

Case reports have suggested that bexarotene may contribute to idiopathic skeletal hyperostosis (Schadt et al, 2011), epidermolysis bullosa (Trufant et, al 2010), and Hodgkin’s lymphoma (AKAY et al, 2010). In addition, bexarotene has been shown to increase clotting times (Hespel et al, 2011).

Dr. Cummings, the sponsor-investigator will hold the IND for bexarotene for this study. Test agent is an approved marketed agent and will be purchased.

- 1. Preclinical Data

1.3.a Bexarotene

In a study involving transgenic mice, Cramer and colleagues (2012) showed that a dose of 100mg/kg of bexarotene induced rapid and substantial reduction of Aβ from the mouse brain. Eleven month old APP-PS1 mice treated with bexarotene for seven days had a 50% reduction in plaque number and significantly reduced levels of soluble and insoluble Aβ. Treatment was also associated with restoration of cognition and memory as measured by the fear conditioning test, the Morris water maze test, nest construction measurements, and olfactory sensory experiments.

The effect of beraxotene appears to be mediated through apolipoprotein E (apoE) mechanisms and the effect on Aß is absent in ApoE null mice. Other mechanisms of action have not been excluded in humans and may involve microglial activation, insulin sensitization, and others.

- 1. Clinical Data to Date

**1.4.a Bexarotene**

Bexarotene is an FDA approved agent for the treatment of CTCL.

Bexarotene capsules were evaluated in 152 patients with advanced and early stage CTCL in two multicenter, open-label, historically-controlled clinical studies conducted in the U.S., Canada, Europe, and Australia.

The advanced disease patients had disease refractory to at least one prior systemic therapy (median of two, range one to six prior systemic therapies) and had been treated with a median of five (range 1 to 11) prior systemic, irradiation, and/or topical therapies. Early disease patients were intolerant to, had disease that was refractory to, or had reached a response plateau of six months on, at least two prior therapies. The patients entered had been treated with a median of 3.5 (range 2 to 12) therapies (systemic, irradiation, and/or topical).

The two clinical studies enrolled a total of 152 patients, 102 of whom had disease refractory to at least one prior systemic therapy, 90 with advanced disease and 12 with early disease. This is the patient population for whom bexarotene capsules are indicated.

Patients were initially treated with a starting dose of 650 mg/m^2^/day with a subsequent reduction of starting dose to 500 mg/m^2^/day. Neither of these starting doses was tolerated, and the starting dose was then reduced to 300 mg/m^2^/day. If, however, a patient on 300 mg/m^2^/day of bexarotene capsules showed no response after eight or more weeks of therapy, the dose could be increased to 400 mg/m^2^/day.

Tumor response was assessed in both studies by observation of up to five baseline-defined index lesions using a Composite Assessment of Index Lesion Disease Severity (CA). This endpoint was based on a summation of the grades, for all index lesions, of erythema, scaling, plaque elevation, hypopigmentation or hyperpigmentation, and area of involvement. Also considered in response assessment was the presence or absence of cutaneous tumors and extra-cutaneous disease manifestations.

All tumor responses required confirmation over at least two assessments separated by at least four weeks. A partial response was defined as an improvement of at least 50% in the index lesions without worsening, or development of new cutaneous tumors or non-cutaneous manifestations. A complete clinical response required complete disappearance of all manifestations of disease, but did not require confirmation by biopsy.

At the initial dose of 300 mg/m^2^/day, 1/62 (1.6%) of patients had a complete clinical tumor response and 19/62 (30%) of patients had a partial tumor response. The rate of relapse (25% increase in CA or worsening of other aspects of disease) in the 20 patients who had a tumor response was 6/20 (30%) over a median duration of observation of 21 weeks, and the median duration of tumor response had not been reached. Responses were seen as early as 4 weeks and new responses continued to be seen at later visits.

Bexarotene has not been used in patients with AD, or at least the experience is not available in the literature. The basis for the use of bexarotene in AD is described above and the approved status of bexarotene and past human experience suggest that it may be used cautiously in this setting to determine its efficacy and safety.

- 1. **Dose Rationale and Risk/Benefits**

**1.5.a Bexarotene**

The dose use in transgenic mice was 100 mg/kg. Using the FDA guidance for estimating the maximum safe starting dose in initial clinical trials for therapeutics in adult healthy volunteers (Center for Drug Evaluation and Research, Pharmacology and Toxicology, July 2005) the calculated dose for use in patients with AD will be approximately 480 mg per day. This is similar to the dose used in CTCL (on average 400 mg per day). Patients with CTCL are likely more compromised from a general health point of view than patients who will be included in a clinical trial of mild to moderate AD. Because of the unknown effect of bexarotene in AD, a dose of 300 mg per day have been chosen and will be assessed in this study. This close to the dose suggested by allometric scaling, allows a margin of improved tolerability, can be administered conveniently, and is less than the dose typically used in cancer settings.

The risk-benefit ratio favors conduct of the clinical trial. AD is an inevitably fatal disease with incremental worsening daily. An agent with known safety parameters used in equivalent doses and in an elderly population that may benefit symptoms and pathophysiology of AD warrants thorough assessment.

No agent has been shown to be effective in slowing the progression of AD. If bexarotene succeeds in modifying the disease process, then the mechanism of action of bexarotene in AD will be studied and novel agents developed. If the mechanism appears to be through RXRs or through ApoE, agents that act on these pathways will become high priority drug candidates. If microglial activation is shown to be the basis for the benefit, then a search for other microglial activators – including less toxic drugs – will be sought. If bexarotene removes Aß and does not improve cognition, it will suggest that the form of Aß removed by bexarotene is not responsible for the cognitive deficit and will enhance our ability to identify optimal targets for further drug development. Information captured in this study may substantially influence the course of AD drug development.

1. STUDY OBJECTIVES

*Primary Objectives*

The primary objective of this study is to determine the safety and biomarker efficacy (based on amyloid imaging) of 300 mg of bexarotene administered for one month and compared to placebo.

*Secondary Objectives*

Secondary objectives include:

- Determine clinical responses after one month double blind therapy on ADAS-cog, CDRSOB, ADCS ADL, NPI.
- Determine amyloid imaging changes after 2 months of active treatment in the bex-bex arm of the open label extension and of one month of treatment in the placebo-bex arm of the open label extension.
- Determine the clinical changes after 2 months of active treatment in the bex-bex arm of the open label extension and of one month of treatment in the placebo-bex arm of the open label extension on ADAS-cog, CDRSOB, ADCS ADL, NPI.
- Collect plasma biomarkers samples at baseline, week 4 and week 8 to provide further insight into the effects of bexarotene (these will include serum Aβ 40 and 42, soluble amyloid precursor protein alpha (sAPP-α), sAPP-β, isoprostanes, cytokines, and others)
- Collect ApoE genotype and conduct analyses to investigate trends toward differential treatment effects by genotype.

1. STUDY DESIGN

3.1 General Design

This is a Phase IIa randomized, double-blind, placebo controlled, parallel group, proof of concept Cleveland Clinic Lou Ruvo Center for Brain Health clinical trial of patients with mild to moderate AD. A single dose of bexarotene will be studied, 300 mg per day given as two 150 mg doses daily. The study will be conducted in a double-blind manner with a 4::1 ratio of randomization of drug to placebo for one month followed by a one month open label treatment. A maximum of twenty patients will be entered into the study. Sixteen will be exposed to bexarotene, and four to placebo for the first month. All patients completing four weeks of treatment will be invited to participate in a four-week open label extension. The primary outcomes of this study are safety and biomarker efficacy as judged by reduction in amyloid plaque burden on amyloid imaging.

Secondary objectives include measurement of clinical outcomes, including cognition, global function, neuropsychiatric symptoms and activities of daily living after 4 and 8 weeks of trial participation. Amyloid imaging at week 8 is also a secondary outcome that will provide information regarding the effects on Aβ of longer term exposure to bexarotene (8 weeks in the group that is exposed to the active agent for the first 4 week and for the 2^nd^ 4 weeks).

- 1. Primary Study Endpoints

The primary study endpoint is a reduction in amyloid burden as measured by standard uptake units regional (SUVR) on amyloid brain imaging obtained through positron emission tomography (PET). The primary efficacy analysis is the difference in amyloid imaging after 1 month.

Another important endpoint is safety. There has been no previous documented exposure of patients with AD to bexarotene and the safety experience in these twenty patients is of paramount importance.

- 1. Secondary Study Endpoints

Secondary study endpoints include measures of cognition (ADAS-cog), global function (CDRSOB), neuropsychiatric symptoms (NPI), and ADLs (ADCS-ADL scale). Changes in drug vs placebo at 1 month and changes in treatment vs baseline at 2 months will be examined. Amyloid imaging will be collected at week 8 and will be compared to baseline and to images collected at week 4. The primary analysis will be conducted after 1 month of treatment.

3.4 Primary Safety Endpoints

Toxicity data will include all adverse events (AEs) and serious adverse events (SAEs) observed in the course of the trial.

Based on the toxicity data available, women of child bearing age will be eliminated from consideration. Serum lipids and cholesterol will be collected at baseline and at two-week intervals. Liver function tests will be collected at baseline and at two-week intervals. TSH and T4 will be collected at baseline and at two-week intervals. CBC will be collected at baseline and at two-week intervals. Slit lamp examinations will be conducted at the beginning of the study and at the end of the planned two-month exposure. Patients will be instructed to take less than 15,000 International Units (IU)/day of vitamin A. Patients will be instructed not to drink grapefruit juice during the course of the study. Patients will be informed of possible photosensitivity and instructed to avoid sun exposure.

The dose of bexarotene used in the transgenic mice experiments was 100mg/day. Based on the FDA guidance for determining human equivalent dosing, the dose of 100mg/day is multiplied by .08 to predict a dose of 8 mg/kg or 480 mg/day for a 60 kilo person. To allow for toxicity in AD, the dosage of 150 mg/twice daily will be used for AD patients.

1. SUBJECT SELECTION AND WITHDRAWAL

4.1 Inclusion Criteria

- Males or females 50 to 90 of age inclusive.
- Diagnosis of probable AD according to National Institute of Neurological and Communicative Disorders and Stroke-Alzheimer’s Disease and Related Disorders Association (NINCDS-ADRDA) criteria.
- Mini-Mental State Examinations (MMSE) score of 10-20.
- If receiving therapy with a cholinesterase inhibitor and/or memantine, the dose of these agents has been stable for at least three months.
- The patient has a research partner willing to participate as part of the study protocol.
- Normal findings at baseline, CBC, chemistry panel, serum lipids, liver functions, TSH, and vitamin B-12.
- Positive amyloid imaging defined as an image read as “abnormal”. Scans will also be graded visually on a 1-4 scale. Abnormal scans will have a score of 3 or higher (Florbetapir FDA Briefing Document, 2011).
- Willing to have ApoE genotype determined (this information will not be disclosed to the patient).
- Females must be postmenopausal.
  1. Exclusion Criteria
- Any clinically relevant neurological disorder capable of producing a dementia syndrome including Parkinson’s disease, stroke, vascular dementia, dementia with Lewy bodies, frontotemporal dementia and others.
- 4 or more micro-hemorrhages (amyloid-related imaging abnormalities – hemorrhage type (ARIA-H) on baseline MRI or any evidence of amyloid-related imaging abnormalities – effusion type (ARIA-E) (Sperling et al, 2011).
- History of malignancy within the past five years with the exception of basal cell or **squamous** cell cancer, in-situ cervical cancer, or localized prostate cancer.
- The subject has any unstable medical illness including hypertension, congestive heart failure, chronic obstructive pulmonary disease, renal failure, liver failure or other organ compromise.
- Other clinically important abnormality on vital signs, physical examination, neurologic examination, laboratory results, or electrocardiogram (ECG) examination (eg. Atrial fibrillation) that could compromise the study or be detrimental to the subject.
- The subject has received bexarotene previously.
- The subject has an allergy to bexarotene.
- The subject has an allergy to a multivitamin.
- Has had a PET scan in the past 12 months.
- Has had radiotherapy in the past year.
  1. Subject Recruitment and Screening

Subjects will be recruited from the Lou Ruvo Center for Brain Health in Las Vegas. Subjects may be referred directly to the clinical trial from community physicians aware of the trial. All advertising material used for the trial will be approved by the Cleveland Clinic Institutional Review Board (IRB) prior to implementation.

**4.4 Early Withdrawal of Subjects**

This is a one month double- blind exposure followed by a one-month open label exposure for those that complete the first month of therapy. The withdrawal of subjects is anticipated to be small given the short duration of planned exposures. Withdrawal can be initiated at the discretion of the trial physician if there is any indication that adverse events emerging in the course of the trial are compromising the patient’s health, cognition, or participation. Following are specific circumstances justifying withdrawal.

- - Sudden disease progression consistent with ARIA-E or microhemorrhages (ARIA-H) and ARIA-E/H confirmed by MRI
  - Development of an inter-current medical condition or need for concomitant treatment that precludes further participation in the trial
  - Unacceptable toxicity or any adverse event that precludes further participation in the trial
  - The investigator removes the patient from the trial in the best interests of the patient
  - Non-adherence to study regimen as determined by pill count
  - Study completion or discontinuation
  - Patient withdraws consent to continued participation in the trial

Patients will be encouraged not to withdraw from the study and any withdrawn patients will be carefully followed to determine both their disease trajectory and to monitor safety.

4.5 Data Collection and Follow Up for Withdrawn Subjects

- - All subjects who discontinue the trial prematurely will be followed.
  - All subjects will have comprehensive contact information for their homes in the communities in which they live.
  - All subjects will have a research partner as a criterion for inclusion in the trial.
  - Every effort will be made to maintain contact with patients discontinuing treatment during the course of the trial.

1. STUDY DRUG

5.1 Description

Each soft gelatin capsule for oral administration contains 75 mg of bexarotene.

The chemical name is 4-[1-(5,6,7,8-tetrahydro-3,5,5,8,8-pentamethyl-2-naphthalenyl) ethenyl] benzoic acid.

Bexarotene is an off-white to white powder with a molecular weight of 348.48 and a molecular formula of C24H28O2. It is insoluble in water and slightly soluble in vegetable oils and ethanol, USP.

Each Targretin (bexarotene) capsule also contains the following inactive ingredients: polyethylene glycol 400, NF, polysorbate 20, NF, povidone, USP, and butylated hydroxyanisole, NF. The capsule shell contains gelatin, NF, sorbitol special-glycerin blend, and titanium dioxide, USP.

Bexarotene will be over-encapsulated with a gelatin capsule to mask the treatment and create identical appearing placebos. Placebo capsule will contain a multivitamin.

5.2 Treatment Regimen

Patients will begin on a dose of one capsule twice daily for the first week and then will be given two capsules twice daily for the remaining three weeks of the double-blind placebo controlled trial. For the open-label continuation of the trial patients will receive two capsules twice daily. Pill counts will be used to assess adherence to this regimen.

5.3 Method for Assigning Subjects to Treatment Regimen

Subjects will be assigned to drug or placebo in a 4 to1 ratio during the month of the double-blind placebo controlled phase of the trial. Patients will receive open-label therapy in the second month of the trial. Randomization will be supervised by the independent study coordinator of Cleveland Clinic Foundation.

- 1. Preparation and Administration of Study Treatment

After receiving the randomized treatments from the research coordinator who will be independent of the research team, study treatment will be provided by a separate research coordinator. Each clinical coordinator involved in this process will receive training regarding the exact dosing and initiation of treatment. Patients will be given 14 capsules, one to take twice per day for the first week of therapy. Patients will then be seen and will be given 28 capsules to be taken as two twice daily for the next week and until the next blood draw. They will be given 56 capsules for the remainder of the double-blind phase of the trial. If they elect to continue in the open label phase of the trial, they will be given 120 capsules for the 4 weeks of the open label treatment.

- 1. Subject Compliance Monitoring

Adherence to the study protocol will be judged by pill counts. Non-adherence in excess of 80% of the anticipated doses will result in study discontinuation.

- 1. Prior and Concomitant Therapy

Patients will be interviewed regarding all concomitant therapy at baseline. Concomitant treatment with a cholinesterase inhibitor or memantine is allowed provided therapy that has been stable for at least three months. Psychotropic medications are also allowed, provided that therapy has been stable for at least one month. Because of a potential interaction between bexarotene and vitamin A, patients will be instructed to take less than 15,000 IU per day of vitamin A. In addition, because bexarotene metabolism can be inhibited by grapefruit or grapefruit juice patients and research partners will be instructed to completely avoid patient consumption of grapefruit, grapefruit juice or other grapefruit products during the course of the study.

Any patient who has had a PET scan in the previous 12 months will be excluded from the study as this might lead to a total radiation exposure beyond allowable limits. Similarly, any patient who has had radiation therapy in the past 12 months will be excluded from the study to limit the total radiation exposure of the patients. The three PET scans proposed as part of this study are within the radiation tolerance human exposure.

Each amyloid PET provides 7mSv of radiation exposure and the accompanying CT has 1 mSv. Each study entails an exposure of 8 mSv. Three scans will total 24 mSv. The maximum exposure allowed is 50 mSv/year.

- 1. Packaging

Patients will be given a bottle of bexarotene capsules containing the specified number of pills (Section 5.4). Patients in this study will receive a maximum of 56 capsules for the final two weeks of the double-blind therapy and 120 capsules for the one month of open-label therapy.

- 1. Blinding of Study Drug (Device or Treatment)

Bexarotene capsules will be over-encapsulated with gelatin capsule covers. There will be identical-appearing placebos which do not contain bexarotene.

- 1. Receiving, Storage, Dispensing and Return
     1. Receipt of Drug Supplies

All drugs for the study will be shipped to the Cleveland Clinic Lou Ruvo Center for Brain Health in Las Vegas, Nevada. Upon receipt of the study treatment supplies, an inventory will be performed and a drug receipt log filled out and signed by the person accepting the shipment. Study staff will count and verify that the shipment contains all the items noted in the shipment inventory. Any damaged or unusable study treatment product in a given shipment will be documented in the study files. The investigator will notify the study sponsor-investigator of any damage or unusable study treatments that were supplied to the investigator site.

- - 1. Storage

Bexarotene can be stored at room temperature. High temperatures will be avoided. The drug will be protected from light. No special temperature controls are necessary beyond those of room temperature.

- - 1. Dispensing of Study Drug

The first one week of drug placebo will be dispensed as one capsule BID for a total of 14 capsules. At the end of one week the patient will be seen and if there is no reason to discontinue therapy, patient will be advanced to two capsules BID and will be given the remaining 28 capsules for the next one week of treatment. On the next visit, 56 capsules for the final two weeks of the double-blind placebo control period will be provided. Patients will be seen at week four and if they elect to continue in the open-label portion of the study they will be given 120 capsules, four tablets per day for 30 days. Regular study drug reconciliation will be performed to document drug assigned, drug consumed, and drug remaining. This reconciliation will be logged on the drug reconciliation form, signed and dated by the study coordinator.

- - 1. Return or Destruction of Study Drug

There will be a final reconciliation of the study drug at the conclusion of the study. The reconciliation will be logged on the drug reconciliation form, signed and dated. Any discrepancies noted will be investigated, resolved and documented prior to return of unused study drug.

- 1. STUDY PROCEDURES
  2. Pre-registration and Screening (Visit 1)

Patients will be screened for a diagnosis of probable AD dementia and will have mild to moderate AD.

Patients will receive baseline laboratory studies, including complete blood count (CBC), chemistry panel, liver function test, TSH, B12, cholesterol, and lipids. These must be shown to be normal prior to randomization. Patients will undergo a checklist to verify the diagnosis of AD dementia and MMSE to ensure they have mild to moderate dementia with MMSE scores 10-20. Patients will have a baseline assessment with ADAS-Cog, CDR, CDRSOB, NPI and ADCS-ADL. MRI obtained in the past and consistent with a diagnosis of AD will be documented.

Baseline MRI will be obtained. Two-dimensional T2*-GRE will be obtained to detect microhemorrhages and T2 FLAIR sequences will be collected to identify ARIA-E. Patients will be excluded from participation if they have 4 or more microhemorrhages or any evidence of ARIA-E.

Amyloid imaging will be obtained. There must be an abnormal scan documenting an amyloid
burden for entry into the study.

ApoE genotype will be collected. Exact genotype (ApoE2,3,4) is not an inclusion/exclusion factor but will be determined and included in the data analysis. Slit lamp examination will be conducted to document the presence of any lens opacifications. This will be done when all other inclusion criteria are met.

Blood will be collected for biomarker measurement. These will include serum Aβ 40 and 42, sAPP-α, sAPP-β, isoprostanes, cytokines and other measures relevant to AD and to bexarotene mechanism of action. In addition, blood will be collected and stored for bexarotene level determination.

6.2 Visit 2 (Baseline, Randomization)

Patients will be randomized within 45 days of baseline screening. At the time of visit 1, all laboratory studies will be reviewed and patients will be randomized only if all laboratories studies are normal. Amyloid imaging must show a positive amyloid. Patients will be given capsules (drug or placebo) for one week of treatment including 14 capsules administered as one capsule BID.

- 1. Visit 3

This visit will occur at the end of one week. It is primarily a safety visit to determine if the patient is able to continue with therapy. If the patient is able to continue, they will be given sufficient medication for an additional one weeks of therapy at two tablets twice daily (total dose 300 mg per day). A comprehensive review of adverse events will be collected at visit two.

- 1. Visit 4

This is a safety visit to determine patient tolerability and check bloods drawn in the past week and to determine lipid levels, liver function test results, TSH, and CBC. If all blood tests are normal or do not require intervention, the patient and care partner will be given 56 capsules to take for the final two weeks of the double blind portion of the study. If the serum lipids are elevated above 3x normal, the patient will be offered treatment with Lipitor. If the patient is receiving Lipitor, the patient will be instructed to increase the dose.

- 1. Visit 5

This visit terminates the double-blind portion of the study. Patients will be assessed with the ADAS-Cog, CDR, CDRSOB, NPI, ADCS-ADL and MMSE. All laboratory studies will be repeated. They will receive amyloid imaging and MRI. Patients will have a slit lamp examination to determine if cataracts are present or changing. Blood will be collected for biomarker measurements. In addition, blood will be collected and stored for bexarotene level determination.

Patients completing the week 4 visit will be invited to participate in a one-month open-label extension study. If they agree to participate in the open-label extension they will be given a one months supply (120 tablets of bexarotene).

Exclusion criteria for participation in the open label extension are identical to those of the double-blind portion of the trial with the exception that 16 of the patients will have been exposed to bexarotene. If any patient has developed 4 or more microhemorrhages in the first month of exposure or has symptomatic amyloid-related imaging abnormalities-effusion type (ARIA-E)(Sperling et al, 2011) they will be excluded from participation in the open label extension.

- 1. Visit 6

On week 8, the patient will have their fifth visit. This will be the termination visit for the open-label extension. At this time patients will have the clinical assessment with ADAS-Cog, CDR, CDRSOB, NPI, ADCS-ADL, and MMSE. All laboratory studies will be repeated. Patients will have a slit lamp examination to determine if cataracts are present or changing and they will have an MRI to assess for ARIA-E and microhemorrhages. Amyloid imaging will be repeated and blood will be collected for biomarker measurements. In addition, blood will be collected and stored for bexarotene level determination.

6.7 Visit 7

Two weeks after the termination of the open-label extension, patients will be reassessed. If any laboratory abnormalities have emerged in the course of the 8-week clinical trial, laboratory measures of that test will be repeated. If abnormalities persist patients will be referred to their principle care physician for treatment of the abnormality.

- 1. Unscheduled Visits

Patients will have unscheduled visits if they report unusual side effects. If the patient reports abrupt worsening of cognition compatible with ARIA-E or microhemorrhage, the patient will be immediately rescanned with MRI.

- 1. Study Calendar of Procedures

| Visit | 1 | 2 | 3 | 4 | 5 | 6 | 7 |
| --- | --- | --- | --- | --- | --- | --- | --- |
| Week | Screening | BL; 0 | 1 | 2 | 4 | 8 | 10 |
| Phase |  | Double blind |  |  | Open label extension |  | 2 week post study follow-up |
| Informed consent | x |  |  |  |  |  |  |
| Demographic data | x |  |  |  |  |  |  |
| Medical history | x |  |  |  |  |  |  |
| Review of medications | x |  |  |  |  |  |  |
| MRI | x |  |  |  | x | x |  |
| Vital signs | x | x | x | x | x | x | x |
| Body weight | x | x | x | x | x | x | x |
| Body height | x |  |  |  |  |  |  |
| Physical and neurological examinations | x |  |  |  |  | x |  |
| ECG | x |  |  |  |  |  |  |
| Hematology and blood chemistry (including lipids and liver functions) | x |  |  | x | x | x |  |
| TSH | x |  |  |  | x | x |  |
| ApoE genotyping | x |  |  |  |  |  |  |
| Slit lamp examination |  | x |  |  | x | x |  |
| Inclusion/exclusion criteria (including MMSE) | x | x |  |  |  |  |  |
| Randomization |  | x |  |  |  |  |  |
| Amyloid imaging | x |  |  |  | x | x |  |
| Serum biomarker measures |  | x |  |  | x | x |  |
| ADAS-cog |  | x |  |  | x | x |  |
| CDR-sob |  | x |  |  | x | x |  |
| NPI |  | x |  |  | x | x |  |
| ADCS-ADL |  | x |  |  | x | x |  |
| MMSE | x | x |  |  | x | x | x |
| Columbia Suicide Severity Rating Scale | x |  |  |  | x | x |  |
| Record adverse events |  |  | x | x | x | x |  |
| Dispense trial medication |  | x | x | x | x |  |  |
| Drug adherence/accountability assessment |  |  | x | x | x | x |  |

- 1. Laboratory Procedures

Laboratory studies will be collected at baseline, at week 2, 4, and at the end of week 8. If any laboratory abnormalities are discovered at week eight, patients will receive follow-up blood studies at week ten. Quest Diagnostics will function as the research reference laboratory and will conduct all laboratory assessments.

1. STATISTICAL PLAN

7.1 Sample Size Determination

This is primarily a safety study to determine the safety of administering bexarotene to individuals with mild to moderate AD. In addition, a biomarker outcome is being collected as a primary endpoint using amyloid imaging. Amyloid reductions in transgenic mice exposed to bexarotene were dramatic - in excess of seventy percent. The natural history of amyloid plaques as seen on amyloid imaging in AD is to remain stable; test-retest reliability is high. Any reduction in excess of test-retest variability (1-4%) will be detectable as a reduction. Such reductions will be apparent in a drug-placebo comparison of 16 drug treated versus 4 placebo treated patients in the double-blind placebo controlled phase of the trial. SUVR comparisons of the before and after scans, as well as the endpoint drug and placebo scans, will be completed.

7.2 Statistical Methods

SUVR analysis comparing baseline versus final scans for bexarotene treated patients and drug versus placebo final scans for bexarotene versus placebo treated patients will be analyzed by Avid on data provided by the Cleveland Clinic. Amyloid imaging analysis will be conducted in conjunction with the Neuroimaging Center of the CCF Neurological Institute (Michael Phillips, MD). The primary analysis will be conducted at the end of the double-blind portion of the study. Secondary analysis will be conducted at the end of the open-label phase of the study.

Drug-placebo differences, as well as baseline end-of-treatment differences, will be analyzed for clinical assessments including ADAS-cog, CDRSOB, NPI, and ADCS-ADL. This study is not expected to be adequately powered to detect clinical differences. ANOVA and MANOVA approaches standard for clinical trial data will be applied. Change in drug-treated compared to changes in placebo-treated patients will be conducted at the end of the double-blind portion of the study. Comparison of baseline vs end of study (8 weeks in the drug-drug treatment group and 4 weeks in the placebo-drug treated group) will be conducted at the end of the 2^nd^ month of the study. Attrition imputation will be conducted through last observation carried forward (LOCF) techniques. Analysis will be conducted in conjunction with Cleveland Clinic Quantitative Health Sciences.

The first step in the analysis will include all patients receiving active treatment compared to all patients on placebo. It is possible that ApoE genotype will influence the effects of bexarotene. Patients will be grouped as treated ApoE 4 carriers vs placebo, treated non-e4 carriers (e3 + e2) vs placebo, and treated ApoE 2 carriers vs placebo for sub-analyses. ApoE 2 is the most uncommon genotype and there may be few or no ApoE 2 carriers in this small sample.

Any changes in statistical methods will be finalized prior to data lock and kept in written (electronic) form. This will be available for review as appropriate.

7.3 Subject Populations for Analysis

A modified intent-to-treat (mITT) analysis will be pursued with all subjects included in the analysis of who are randomized and received at least one dose of treatment. LOCF will be applied to impute values lost through attrition.

In addition, a protocol compliant population analysis will include patients who are randomized and received the protocol-required study drug exposures.

**7.4 Amyloid Imaging Analysis**

Amyloid scans will be graded on a 0 to 4 basis based on a grey scale template. Readers will be blind to treatment assignment. Differences in the treatment group from baseline to week 4 will be compared with difference in the placebo group from baseline to week 4.

In addition, a semiquantitative SUVR analysis will be obtained in 6 cortical regions in analyses conducted by Avid. Differences in the treatment group from baseline to week 4 will be compared with difference in the placebo group from baseline to week 4. This semiquantitative analysis will comprise the primary efficacy readout of the study. The analysis will be conducted in conjunction with the Neuroimaging Center of the CCF Neurological Institute (M Phillips, MD, Director).

1. SAFETY AND ADVERSE EVENTS

8.1 Definitions

International Conference of Harmonization (ICH) guidelines define an AE as any medical occurrence in a patient or clinical investigation subject administered a pharmaceutical product and which does not necessarily have to have a causal relationship with this treatment. An AE can therefore be any unfavorable and unintended sign (including an abnormal laboratory finding, for example) symptom, or disease temporarily associated with the use of a medicinal product whether or not considered related to this medicinal product.

A SAE is any medical occurrence or affect that at any dose:

- Results in death
- Is life threatening
- Requires hospitalization or prolongation of existing inpatient hospitalization
- Results in persistent or a significant disability or incapacity
- Is a congenital anomaly or birth defect
- Is cancer
- Life threatening in the definition of a SAE refers to an event in which the subject was at risk of death at the time of event; it does not refer to an event which hypothetically might have caused death if it were more severe.

8.2 Recording of Adverse Events

At each contact with the subject, the investigator will seek information on AEs by specific questioning and, as appropriate, by examination. Information on all adverse events will be recorded immediately in the source document, and also in the appropriate AE section of the case report form (CRF). All clearly related signs, symptoms, and abnormal diagnostic procedures results will also be recorded in the source document.

The clinical course of each event will be followed until resolution, stabilization, or until it has been determined that the study treatment or participation is not the cause. Serious adverse events that are still ongoing at the end of the study period will be followed up to determine the final outcome. Any SAE that occurs after the study period and is considered to be possibly related to the study treatment or study participation will be recorded and reported immediately.

8.3 Reporting of Serious Adverse Events

8.3.1 Study Sponsor-Investigator Notification by Investigator

A SAE will be reported to the study sponsor-investigator by telephone within 24 hours of the event. An SAE form will be completed by the investigator and faxed to the study sponsor within 24 hours. The investigator will keep a copy of this SAE form on file at the study site. Report a SAE by phone and facsimile to Michelle Sholar, 702-483-6026, fax 702-483-6028.

At the time of the initial report the following information will be provided:

- Study identifier
- Study Center
- Subject number
- A description of the event
- Date of onset
- Current status
- Whether study treatment was discontinued
  - The reason why the event is classified as serious
  - Investigator assessment of the association between the event and study treatment

Within the following 48 hours, the investigator will provide further information on the AE in the form of a written narrative. This will include a copy of the completed SAE form and any other diagnostic information that will assist the understanding of the event. Significant new information on ongoing SAEs will be provided promptly to the study sponsor-investigator.

- - 1. IRB Notification by Investigator

Reports of all SAEs (including follow-up information) will be submitted to the Cleveland Clinic Foundation (CCF) IRB per the guidelines of the CCF IRB Standard Operating Procedures. Copies of each report and documentation of IRB notification and response will be filed in the regulatory binder.

The following four types of events will be reported to the IRB (these follow the Case Cancer IRB because this is an anti- cancer agent being used in patients with mild to moderate AD):

1. Adverse events which are serious, unexpected, and related or possibly related to participation in the research.

2. Serious adverse events that are expected in some subjects, but are determined to be occurring at a significantly higher frequency or severity than expected.

3. Other unexpected adverse events, regardless of severity, that may alter IRB analysis of the risk versus potential benefit of the research and, as a result, warrant consideration of substantive changes in the research protocol or informed consent process/document.

4. Unanticipated Problems involving risks to subjects or others or any serious or continuing noncompliance with this policy or the requirements or determinations of the IRB.

8.3.3 FDA Notification by Sponsor-Investigator

We will notify the FDA by telephone or by facsimile transmission of any unexpected fatal or life threatening experience associated with the use of the drug as soon as possible, but no later than seven calendar days from our original receipt of the information.

If a previous adverse event that was not initially deemed reportable is later found to fit the criteria for reporting, the study sponsor-investigator will submit the adverse event in a written report to the FDA as soon as possible, but no later than 15 calendar days from the time the determination is made.

- 1. Unblinding Procedures

The research coordinator will inform the sponsor-investigator of all subjects whose treatment was unblinded with 24 hours of unblinding. Most unblinding will be part of managing an SAE and will be reported with the SAE. Unblinding that was not associated with an SAE will be reported in a timely manner. This will be done within 24 hours by telephone or facsimile, and will be followed by a written narrative of the reason for unblinding within 24 hours of the event.

- 1. Stopping Rules

If 4 or more patients worsen clinically in a manner greater than expected for untreated AD, the protocol will be stopped. Similarly if 4 or more patients show ARIA-E or microhemorrhages on the 2^nd^ MRI, the protocol will be stopped.

- 1. Medical Monitoring

The principal investigator will oversee the safety of patients in the bexarotene study. The safety monitoring will include careful assessment and appropriate reporting of adverse events as noted above as well as the construction and implementation of a site data and safety monitoring plan. Medical monitoring will include a regular assessment of the number and type of SAEs. Medical monitoring for the trial would be done by Dylan Wint, MD, of the Lou Ruvo Center for Brain Health.

8.6.1 Internal Data Safety Monitoring Board

A data safety monitoring committee is not deemed necessary for this twenty patient initial study of bexarotene. .

- 1. DATA HANDLING AND RECORD KEEPING
  2. Confidentiality and Privacy

Information about study subjects will be kept confidential and managed according to the Health Insurance Portability and Accountability Act of 1996 (HIPAA). Subjects or a legally acceptable surrogate will provide authorization that they have been informed of the following:

- What protected health information (PHI) will be collected from subjects in this study
- Who will have access to that information and why
- Who will use or disclose that information
- The rights of a research subject to revoke authorization for use of the PHI.

The HIPAA language is included as part of the informed consent form.

- 1. Source Documents

Source documentation for all entry criteria will be available in the patient’s research record. These records will be retained as required by law.

- 1. Case Report Forms

The study CRFs have been constructed for this study (see attachments; section 15). All data will be collected on the CRF and all missing data on the CRF will be explained.

- 1. Records Retention

All documents will be retained for a minimum period of two years following completion of the study.

**9.5 Database**

The database will be constructed by CCF Quantitative Health Sciences (QHS). The database will be populated by the study research coordinator.

1. STUDY MONITORING, AUDITING AND INSPECTING
   1. Study Monitoring Plan

This study will be monitored according to the monitoring plan in Attachment 15. The investigator will allocate adequate time for such monitoring activities. The Investigator will also ensure that the monitor or other compliance or quality assurance reviewer is given access to all the above noted study-related documents and study related facilities and has adequate space to conduct the monitoring visit.

The very short nature of this study (two months of exposure per patient) and the small size of the study (twenty patients) suggest that monitoring at month one and study completion will be adequate.

- 1. Auditing and Inspecting

The investigator will permit study-related monitoring, audits, and inspections by the IRB, the sponsor-investigator, government regulatory bodies, and institutional compliance and quality assurance groups of all related documents (for example, source documents, regulatory documents, data collection instruments, study data etc.). The investigator will ensure the capability for inspections of applicable study-related facilities. Participation as an investigator in this study implies acceptance of potential inspection by government regulatory authorities and applicable Institutional compliance and quality assurance offices.

1. ETHICAL CONSIDERATIONS

This study will be conducted according to US and international standards of Good Clinical Practice (GCP)(FDA Title 21 part 312 and ICH guidelines), applicable government regulations and Institutional research policies and procedures.

This protocol and any amendments will be submitted to the IRB, in agreement with local legal prescriptions, for formal approval of the study conduct. The decision of the IRB concerning the conduct of the study will be made in writing to the investigator and a copy of this decision will be provided to the sponsor-investigator before commencement of this study. The investigator will have a list of IRB members and their affiliates.

All subjects for this study will be provided a consent form describing this study and providing sufficient information for subjects or legally acceptable surrogates to make an informed decision

1. STUDY FINANCES
   1. Funding Source

This study is being funded by donors who support the research activities of the Cleveland Clinic Lou Ruvo Center for Brain Health. Funders did not participate in the design of the study and do not have any financial interest in the outcome of the study.

- 1. Conflict of Interest

Any investigator who has a conflict of interest with this study will have the conflict reviewed the Conflict of Interest Committee with a Committee-sanctioned conflict management plan that has been reviewed and approved prior to participation in this study. All Cleveland Clinic investigators will follow the Institutional conflict of interest policy.

- 1. Subject Stipends or Payments

No subject stipend or payment is anticipated for this study.

1. PUBLICATION PLAN

Dr. Jeffrey L. Cummings, principle investigator, has primary responsibility for publication of the results of this study. It is the full intention of the investigator to publish the results of this study as soon as possible. Neither the complete study nor any part of the results of the study carried out under this protocol, nor any of the information provided by the sponsor- investigator for the purposes of performing the study, will be published or passed on to any third party without the consent of the study sponsor- investigator. Any investigator involved with this study is obligated to provide the sponsor- investigator with complete test results and all data derived from the study.

1. REFERENCES
   1. Akay BN, Ozcan M, Sanli H. Development of Hodgkin’s lymphoma under bexarotene treatment for Sezary syndrome and review of the literature. Journal of Drugs in Dermatology 2010;9.8:1014
   2. Albert MS, DeKosky ST, Dickson D, et al. The diagnosis of mild cognitive impairment due to Alzheimer’s disease: recommendations from the National Institute on Aging-Alzheimer’s Association workgroups on diagnostic guidelines for Alzheimer’s disease. Alzheimer’s Dement 2011; 7(3):270-279.
   3. Alzheimer’s disease facts and figures. Alzheimers Dement 2012;8:131-168.
   4. Burke WJ, Miller JP, Rubin EH, et al. Reliability of the Washington University clinical dementia rating. Arch Neurol 1988;45(1):31-32.
   5. Clark CM, Schneider JA, Bedell BJ, Beach TG, Bilker WB, Mintun MA, Pontecorvo MJ, Hefti F, Carpenter AP, Flitter ML, Krautkramer MJ, Kung HF, Coleman RE, Doraiswamy PM, Fleisher AS, Sabbagh MN, Sadowsky CH, Reiman PE, Zehntner SP, Skovronsky DM. JAMA 2011;305(3):275-283.
   6. Cramer PE, Cirrito JR, Wesson DW, Lee CY, Karlo JC, Zinn AE, Casali BT, Restivo JL, Goebel WD, James MJ, Brunden KR, Wilson DA, Landreth GE. ApoE-directed therapeutics rapidly clear β-amyloid and reverse deficits in AD mouse models. Science 2012;335:1503-1506.
   7. Cummings JL, Mega M, Gray K, Rosenberg-Thompson S, Carusi DA, Gornbein J. The neuropsychiatric inventory: comprehensive assessment of psychopathology in dementia., Neurology 1994;44:2308-2314.
   8. Dubois B, Feldman HH, Jacova C, et al. Research citeria for the diagnosis of Alzheimer’s disease: revising the NINCDS-ADRDA critieria. Lancet Neurol 2007; 6(8):734-746. Review.
   9. Dubois B, Feldman HH, Jacova C, et al. Revising the definition of Alzheimer’s disease: a new lexicon. Lancet Neurol 2010; 9(11):1118-1127.
   10. Florbetapir FDA Advisory Committee Briefing Document 2011. NDA 202-008.
   11. Galasko D, Bennett D, Sano M, et al. An inventory to assess activities of daily living for clinical trials in Alzheimer’s disease. The Alzheimer’s Disease Cooperative Study. Alzheimer Dis Assoc Disord 1997;11(suppl 2):S33-39.
   12. Goodman AB. Retinoid receptors, transporters, and metabolizers as therapeutic targets in late onset Alzheimer disease. J Cell Physiol 2006;209:598-603.
   13. Hespel A, Yous S, Charef S. Interactions of bexarotene (LGD1069, Targretin) with the coagulation system. Cancer Chemother Pharmacol 2011;68:847-854
   14. Huang Y, Mucke L. Alzheimer mechanisms and therapeutic strategies. Cell 2012;148:1204-1222.
   15. Hughes CP, Berg L, Danziger WL, Coben LA, Martin RL. A new clinical scale for the staging of dementia. Br J Psychiatry 1982;140:566-572.
   16. Ittner L M, Gotz J. Amylid-β and tau – a topic pas de leux in Alzheimer’s disease. Nature Reviews 2011;12:67.
   17. Jarvis CI, Goncalves MB, Clarke E, Dogruel M, Kalindjian SB, Thomas SA, Maden M, Corcoran JPT. Retinoic acid receptor-α signaling antagonizes both intracellular and extracellular amyloid-β production and prevents neuronal cell death caused by amyloid-β. European Journal of Neuroscience 2010;32:1246-1255.
   18. Liang Y, Lin S, Beyer TP, Zhang Y, Wu X, Bales KR, DeMattos RB, May PC, Li SD, Jiang XC, Eacho PI, Cao G, Paul SM. A liver X receptor and retinoid X receptor heterodimer mediates apolipoprotein E expression, secretion and cholesterol homeostasis in astrocytes. J Neurochem 2004;88:623-634.
   19. Lukiw WJ. Amyloid beta (Aβ) peptide modulators and other current treatment strategies for Alzheimer’s disease (AD). Expert Opin Emerging Drugs 2012;17:43-60.
   20. Lynch CA, Walsh C, Blanco A, et al. The clinical dementia rating sum of box score in mild dementia. Dement Geriatr Cogn Disord 2006;21(1):40-43.
   21. Massoud F, Leger GC. Pharmacological treatment of Alzheimer disease. Can J Psychiatry 2011;56:579-88.
   22. McKhann G, Drachman D, Folstein M, Katzman R, Price D, Stadlan EM. Clinical diagnosis of Alzheimer’s disease: report of the NINCDS-ADRDA Work Group under the auspices of Department of Health and Human Services Task Force on Alzheimer’s Disease. Neurology 1984; 34(7):939-944.
   23. McKhann GM, Knopman DS, Chertkow H, et al. The diagnosis of dementia due to Alzheimer’s disease: recommendations from the National Institute on Aging-Alzheimer’s Association workgroups on diagnostic guidelines for Alzheimer’s disease. Alzheimer’s Dement 2011; 7(3):263-269.
   24. Morris JC, Ernesto C, Schafer K, et al. Clinical dementia rating training and reliability in multicenter studies: the Alzheimer’s disease cooperative study experience. Neurology 1997;48(6):1508-1510.
   25. Mukherjee R, Davies PJA, Crombie DL, Bischoff ED, Cesario RM, Jow L, Hamann LG, Boehm MF, Mondon CE, Nadzan AM, Paterniti JR, Heyman RA. Sensitization of diabetic and obese mice to insulin by retinoid X receptor agonists. Nature 1997;386:407-410.
   26. Rosen WG, Mohs RC, Davis KL. A new rating scale for Alzheimer’s disease. Am J Psychiatry 1984;141:1356-1364.
   27. Rowe CC, Villemagne VL. Brain amyloid imaging. J Nucl Med 2011;52(11):1733-1740.
   28. Schadt CR, Zic JA, Fuchs HA. Diffuse idiopathic skeletal hyperostosis associated with bexarotene. J AM Acad Dermatol 2011;65:883-884.
   29. Sperling RA, Aisen PS, Beckett LA, et al. Toward defining the preclinical stages of Alzheimer’s disease: recommendations from the National Institute on Aging-Alzheimer’s Association workgroups on diagnostic guidelines for Alzheimer’s disease. Alzheimers Dement 2011; 7(3):280-292.
   30. Sperling RA, Jack CR Jr., Black SE et al. Amyloid-related imaging abnormalities in amyloid-modifying trials: Recommendations from the Alzheimer’s Association Research Roundtable Workgroup. Alzheimer’s Dem 2011; 7: 367-385.
   31. Suon S, Zhao J, Villarreal SA, Anumula N, Liu M, Carangia LM, Renger JJ, Zerbinatti CV. Systemic treatment with liver X receptor agonists raises apolipoprotein E, cholesterol , and amyloid-β peptides in the cerebral spinal fluid of rats. Molecular Neurodegeneration 2010;5:44.

# Thies W, Bleiler L. 2011 Alzheimer’s diease facts and figures. Alzheimers Dement 2011; 7:208-244.

- 1. Tippmann F, Hundt J, Schneider A, Endres K, Fahrenholz F. Up-regulation of the α-secretase ADAM10 by retinoic acid receptors and acitretin. FASEB J 2009:23;1643-1654.
  2. Trufant JW, Kreizenbeck GM, Carlson KR, et al. A transient epidermolysis bullosa simplex-like phenotype associated with bexarotene treatment in a G138E KRT5 heterozygote. J Cutan Pathol 2010;37:1155-1160
  3. Yokokura M, Mori N, Yagi S, Yoshikawa E, Kikuchi M, Yoshihara Y, Wakuda T, Sugihara G, Takebayashi K, Suda S, Iwata Y, Ueki T, Tsuchiya KJ, Suzuki K, Nakamura K, Ouchi Y. In vivo changes in microglial activation and amyloid deposits in brain regions with hypometabolism in Alzheimer’s disease. Eur J Nucl Med Mol Imaging 2011;38:343-351.

1. ATTACHMENTS

- Sample Consent Form
- Case Report Forms
- Rating Scales
- Study Procedures Flowchart/Table
- Monitoring Plan
- Bexarotene Investigators Brochure

**Study Procedure Flowchart/Table**

| Visit | 1 | 2 | 3 | 4 | 5 | 6 | 7 |
| --- | --- | --- | --- | --- | --- | --- | --- |
| Week | Screening | BL; 0 | 1 | 2 | 4 | 8 | 10 |
| Phase |  | Double blind |  |  | Open label extension |  | 2 week post study follow-up |
| Informed consent | x |  |  |  |  |  |  |
| Demographic data | x |  |  |  |  |  |  |
| Medical history | x |  |  |  |  |  |  |
| Review of medications | x |  |  |  |  |  |  |
| MRI | x |  |  |  | x | x |  |
| Vital signs | x | x | x | x | x | x | x |
| Body weight | x | x | x | x | x | x | x |
| Body height | x |  |  |  |  |  |  |
| Physical and neurological examinations | x |  |  |  |  | x |  |
| ECG | x |  |  |  |  |  |  |
| Hematology and blood chemistry (including lipids and liver functions) | x |  |  | x | x | x |  |
| TSH | x |  |  |  | x | x |  |
| ApoE genotyping | x |  |  |  |  |  |  |
| Slit lamp examination |  | x |  |  | x | x |  |
| Inclusion/exclusion criteria (including MMSE) | x | x |  |  |  |  |  |
| Randomization |  | x |  |  |  |  |  |
| Amyloid imaging | x |  |  |  | x | x |  |
| Serum biomarker measures |  | x |  |  | x | x |  |
| ADAS-cog |  | x |  |  | x | x |  |
| CDR-sob |  | x |  |  | x | x |  |
| NPI |  | x |  |  | x | x |  |
| ADCS-ADL |  | x |  |  | x | x |  |
| MMSE | x | x |  |  | x | x | x |
| Columbia Suicide Severity Rating Scale | x |  |  |  | x | x |  |
| Record adverse events |  |  | x | x | x | x |  |
| Dispense trial medication |  | x | x | x | x |  |  |
| Drug adherence/accountability assessment |  |  | x | x | x | x |  |
