# Supplementary material for: Double-blind, placebo-controlled, proof-of-concept trial of bexarotene Xin moderate Alzheimer’s disease
Source: Alzheimers Res Ther. 2016 Jan 29;8:4. doi: 10.1186/s13195-016-0173-2 (PMC4731943; doi:10.1186/s13195-016-0173-2)
Supplement: Additional file 2: — BEATADStatisticalPlanFinal_1 3_20141209. (DOC 467 kb) [file 13195_2016_173_MOESM2_ESM.doc]

**Phase II Study Evaluating the Safety and Biomarker Efficacy of Bexarotene in Patients with Mild to Moderate Alzheimer’s Disease:**

**BExarotene Amyloid Treatment for Alzheimer’s Disease (BEAT AD)**

**Statistical Analysis Plan, Version 1.3**

James Bena, M.S., Biostatistician

The Cleveland Clinic Foundation

Final Version: December 9, 2014

**FINAL**

**Table of Contents**

1. Study Synopsis 4

1.1 Protocol Version 4

1.2 Study Description 4

2 Primary Objectives 4

2.1 Group Comparisons of Changes of Clinical Measures at 4 Weeks 4

3 Secondary Objectives 4

3.1 Secondary Group Comparisons of Changes in Amyloid Burden 4

3.2 Group Comparisons of Changes of Clinical Measures at 4 Weeks 4

3.3 Within Group Changes at 8 Weeks for Bexarotene Treated Group 5

3.4 Group Comparisons and Within Group Changes on Serum and Plasma Biomarkers 5

3.5 Descriptive Summaries of Safety Endpoints and Cholesterol Elevation 5

4 Planned Exploratory Objectives 5

4.1 Associations between Cholesterol Change and Clinical Measures 5

4.2 Associations between Changes in Amyloid Burden with Changes in Clinical Measures 5

4.3 Associations between Cholesterol Change and Changes in Amyloid Burden 5

5 General Analysis Considerations 5

5.1 Populations of Interest 5

5.2 Analysis Data Set 5

5.3 Descriptive Methods 6

5.4 Software 6

6 Planned Analysis 6

6.1 Demographic, Medical History, and Baseline Characteristics 6

6.2 Analysis of Primary Objectives 6

6.3 Analysis of Secondary Objectives 6

6.4 Analysis of Planned Exploratory Analysis 6

7 Subgroup Analyses 7

8 Further Exploratory Data Analysis 7

9 Study Flow Consort Diagram

10 Table Descriptions 7

10.1 Baseline Characteristics 7

10.2 Primary Outcome Results 7

10.3 Secondary Outcome Results 7

10.4 Planned Exploratory Analyses 8

10.5 Supplementary Tables 8

11 Table and Figure Shells 9

11.1 Baseline Characteristics 9

11.2 Primary Outcome Results 10

11.3 Secondary Outcome Results 11

11.4 Planned Exploratory Analyses 26

11.5 Supplementary Tables 30

A.1 Appendix 1: Summary of Statistical Analysis Plan Changes 31

**1. Study Synopsis**

1.1 Protocol Version

This document describes the statistical analysis that will be employed to evaluate the outcomes of success described in the protocol version October 23, 2012. This document is based upon the descriptions of the statistical analysis of the secondary endpoints described on page 17 of that document and is based upon the statistical plan described on pages 36 through 38.

1.2 Study Description (summarized from section 3 of the protocol)

This is a Phase IIa randomized, double blind, placebo controlled, parallel group, proof of concept single site study, to evaluate the safety and biomarker efficacy of Bexarotene in patients with mild to moderate AD.

The study consists of three phases: 4 week double blind placebo controlled period followed by 4 week open label extension, with a two week post study follow up period.

The double blind eligible patients will be randomized with a 4:1 ratio at baseline to receive bexarotene or matching placebo. The study drug will be given as one capsule of 75 mg twice daily on the first week then increasing the dosage up to two capsules of 75 mg twice daily orally on the 2nd week up to the open label phase. A total of 20 subjects will be enrolled: 16 will receive Bexarotene and 4 will receive matching placebo for the first 4 weeks. Upon the completion of 4 week double blind treatment, subjects will enter into an open label extension phase in which all subjects will receive bexarotene (150 mg twice daily [150 mg bid]). All study medications including Bexarotene and placebo capsules are of identical appearance in order to maintain the integrity of the blind.

**2. Primary Objectives**

2.1. Group Comparisons of Changes in Amyloid Burden

To evaluate the efficacy of Bexarotene 150 mg bid compared to placebo on the reduction of amyloid burden after 4 weeks of double blind study treatment. This analysis will be based on the calculations using the composite region using the white matter standard.

**3. Secondary Objectives**

3.1 Secondary Group Comparisons of Changes in Amyloid Burden

The efficacy of Bexarotene 150 mg bid compared to placebo after 4 week treatment on amyloid burden will be performed by comparing amyloid burden changes regionally using all subjects, and then among e4 carriers (overall and among heterozygote and homozygote groups, if sample size permits) and non e4 carriers. The change in the number judged to be positive will also be summarized. All previous analyses will then be repeated using the alternative cerebellum standard.

3.2. Group Comparisons of Changes of Clinical Measures at 4 Weeks

The efficacy of Bexarotene 150 mg bid compared to placebo after 4 week treatment on cognition (ADAS-Cog, MMSE), global function (CDR-SOB), neuropsychiatric symptoms (NPI) and activities of daily living (ADCS-ADL) will be measured using the change from baseline. Analyses of each endpoint will be performed overall, and if the distribution of e4 carriers permits, stratified by e4 carrier status. This includes an overall analysis of e4 carriers and separate analyses may also be performed within heterozygotes and homozygote groups.

3.3. Within Group Changes at 8 Weeks for Bexarotene Treated Group

Analysis will be performed using the group that is randomized to receive the active treatment (Bexarotene 150 mg bid) at baseline and continue on Bexarotene during the open label study phase. The changes in these subjects on amyloid burden (using both definition standards), cognition (ADAS-Cog, MMSE), global function (CDR-SOB), neuropsychiatric symptoms (NPI) and activities of daily living (ADCS-ADL) will be measured between baseline and 8 weeks. Trends in amyloid burden change will be described using at baseline, 4 weeks, and 8 weeks. Analyses will be performed using all subjects and then stratified by e4 carrier status (overall e4 carriers, heterozygote e4, and homozygote e4, non-carrier groups), if feasible.

3.4 Group Comparisons and Group Changes on Serum and Plasma Biomarkers

Changes in serum and plasma biomarkers between the Bexarotene 150mg bid group and Placebo groups from baseline to 4 weeks will be performed using all subjects and then stratified by e4 carrier status (overall e4 carriers, heterozygote e4, and homozygote e4, non-carrier groups), if feasible. Within the group receiving the active treatment (Bexarotene 150 mg bid) at baseline and continuing on Bexarotene during the open label study phase, change from baseline to 8 weeks will also be estimated overall and then by e4 carrier status.

3.5. Descriptive Summaries of Safety Endpoints and Cholesterol Elevation

Group summaries of cholesterol elevation to 3 or 10 times normal levels, proportion of patients with adverse events, serious adverse events, laboratory abnormalities, and EKG abnormalities, and summaries of the frequency of such events will be summarized.

**4. Planned Exploratory Objectives**

4.1 Associations between Cholesterol Change and Clinical Measures

Associations between changes at 4 weeks in cholesterol and changes seen in cognition (ADAS-Cog, MMSE), global function (CDR-SOB), neuropsychiatric symptoms (NPI) and activities of daily living (ADCS-ADL) using all subjects will be evaluated.

4.2. Associations between Changes in Amyloid Burden with Changes in Clinical Measures

Associations between changes at 4 weeks in amyloid burden and changes seen in cognition (ADAS-Cog, MMSE), global function (CDR-SOB), neuropsychiatric symptoms (NPI) and activities of daily living (ADCS-ADL) among all subjects will be evaluated.

4.3 Associations between Cholesterol Change and Changes in Amyloid Burden

Associations between changes at 4 weeks in cholesterol and changes seen in amyloid burden among all subjects will be evaluated.

**5. General Analysis Considerations**

5.1. Study Populations

- The Intent-to-Treat Population will include all consented, randomized subjects grouped according to their assigned treatment group.
- The modified Intent-to-Treat Population will include all consented and randomized subjects who receive at least one dose of their assigned treatment.
- The Per-Protocol Population will include all patients who complete the 4 week study endpoint and have demonstrated adherence to assigned treatment of at least 80% of anticipated doses.

5.2 Analysis Data Set

The primary analysis of efficacy endpoints will be evaluated using the modified intent-to-treat population. Safety endpoints will use the intent to treat population.

5.3 Descriptive Methods

Summaries of the distributions for the endpoints and other collected measures will be created using frequencies and percentages for categorical factors, and mean and standard deviation for continuous measures. Specific percentiles may also be used to describe continuous distributions if the data is highly skewed, or exhibit other characteristics of non-normality.

5.4 Software

Statistical tests will be performed using SAS software, although other statistical resources such as R or StatXact may also be used as needed.

**6. Planned Analysis**

6.1 Demographic, Medical History, and Baseline Characteristics

In order to describe the cohort, information regarding age, gender, medical history, and baseline health measures will be summarized for the entire cohort and by treatment group. In keeping with CONSORT guidelines for clinical trials, formal statistical testing of the group differences will not be performed.

6.2 Analysis of Primary Objectives

Changes in amyloid burden measures at 4 weeks between treatment groups overall, based on the white matter standard definition of amyloid burden will be performed using two-sample t-tests. If the distributions of responses are not normally distributed, logarithmic transformations of the measurements will be performed prior to statistical testing. Data will then be transformed back to the original scale and presented as geometric means in tables and figures.

6.3 Analysis of Secondary Objectives

Changes in continuous measures at 4 weeks between treatment groups will be performed using two-sample t-tests. Given the small sample size, if distributional assumptions are not met, logarithmic transformations will be performed prior to analysis. Changes at 8 weeks will be evaluated using linear mixed effect models. Specific contrasts will be defined to test changes within group at 8 weeks. Estimated levels of each measure at baseline, 4 weeks, and 8 weeks will also be presented. Descriptive summaries of cholesterol elevation and safety endpoints will be created using frequencies and percentages. Mean cholesterol levels will also be summarized by group and time point. Comparisons between groups on the frequency of adverse events will be performed using Fisher exact tests. All inferences will be performed at the 0.05 level.

If clinically important differences in baseline MMSE, florbetapir, education, or age exist, adjusted analyses may be performed. Given the small sample size, these factors may be analyzed separately, or if included concurrently, penalized regression methods will be used to control for optimism in the analysis.

Missing data at 4 weeks are not expected, but if missing data do arise, the use of maximum likelihood methods such as mixed effect models will be used to account for missing data.

6.4 Analysis of Planned Exploratory Analysis

Associations among continuous change measures of cognitive burden, amyloid burden, and cholesterol levels will be performed using Spearman correlation, given the ordinal nature of the burden measure and expected non-normality of cholesterol change. Graphical scatterplots of these relationships will be created and smooth functions (i.e. LOESS curves) will be used to show patterns. Tests of the Spearman correlations against a null hypothesis of no relationship will be performed at the 0.05 significance level.

**7. Subgroup Analyses**

The above analyses will be repeated stratifying by e4 carrier status, if feasible, statistically. Methods used will be similar to the above approaches.

A per-protocol analysis of the same outcomes is planned using those who completed the assigned therapy as defined by randomization.

**8. Further Exploratory Data Analysis**

Graphs and data summaries will be used to evaluate the structure of the data, and to explore the relationships among baseline characteristics and between these characteristics and outcome measures. Boxplots, spaghetti plots, and scatterplots may be used to assess the relationship of variables at fixed time points or across time.

**9. Study Flow Consort Diagram**

A description of participant participation per the CONSORT guidelines will be developed. The diagram will describe the cohort status of the study from screening to completion. Group summaries at each time point following randomization will be shown.

- Number of subjects screened
- Number and reasons for screening failures
- Number of subjects randomized
- Number of subjects who completed the Week 4 assessments
- Number of subjects who completed the Week 8 assessments
- Number and reasons for subjects who discontinued the study before Week 8

**10. Table Descriptions**

10.1 Baseline Characteristics

Table 1. Summaries of baseline characteristics overall and by group.

Figure 1. CONSORT Diagram flow chart showing subject participation in the study.

10.2. Primary Outcome Results

Table 2. Comparisons of mean changes from baseline to 4 weeks in amyloid burden between treatment groups.

Figure 2. Boxplots showing changes from baseline to 4 weeks in amyloid burden by treatment group.

10.3. Secondary Outcome Results

Table 3. Comparisons of mean changes from baseline to 4 weeks in amyloid burden between treatment groups.

Table 4. Comparisons of changes at 4 weeks in clinical measures between treatment groups.

Table 5. Evaluations of changes at 8 weeks in clinical measures in the group receiving Bexarotene 150mg bid at baseline and the open label phase.

Table 6. Comparisons of changes at 4 weeks in amyloid burden between treatment groups (alternative standard).

Table 7. Comparisons of changes at 4 weeks in serum and plasma biomarkers between treatment groups.

Table 8. Evaluations of changes at 8 weeks in serum and plasma biomarkers in the group receiving Bexarotene 150mg bid at baseline and the open label phase.

Table 9. Summaries of cholesterol elevation and safety endpoints overall and by group through 4 weeks.

Table 10. Summaries of mean cholesterol levels by group over time are shown.

Figure 3. Line plot showing cholesterol levels by subject over time are shown.

Table 11. Summaries of safety endpoints overall and by group during the study.

10.4. Planned Exploratory Analyses

Table 12. Spearman correlations between changes at 4 weeks among clinical measures, cholesterol levels, and amyloid burden.

Figure 4. Scatterplots showing relationships between cholesterol level changes and clinical measure changes.

Figure 5. Scatterplots showing relationships between amyloid burden changes and clinical measure changes.

Figure 6. Scatterplots showing relationships between amyloid burden changes and cholesterol level changes.

10.5 Supplementary Tables

Table S1. Per protocol comparisons of changes at 4 weeks in amyloid burden measures between treatment groups (see Table 3).

Table S2. Per protocol comparisons of changes at 4 weeks in amyloid burden measures between treatment groups (alternative standard) (see Table 6).

Table S3. Per protocol comparisons of changes at 4 weeks in clinical measures between treatment groups (see Table 4).

Table S4. Per protocol evaluations of changes at 8 weeks in clinical measures in the group receiving Bexarotene 150mg bid at baseline and the open label phase (see Table 5).

Table S5. Adjusted comparisons of changes at 4 weeks in amyloid burden measures between treatment groups (see Table 3).

Table S6. Adjusted comparisons of changes at 4 weeks in amyloid burden measures between treatment groups (alternative standard) (see Table 6).

Table S7. Adjusted comparisons of changes at 4 weeks in clinical measures between treatment groups (see Table 4).

Table S8. Adjusted evaluations of changes at 8 weeks in clinical measures in the the group receiving Bexarotene 150mg bid at baseline and the open label phase (see Table 5).

**11. Table and Figure Shells**

11.1 Baseline Characteristics

Table 1. Summaries of baseline characteristics overall and by group.

|  | **Total** | | **Bexrotene**  **(N = 16)** | **Control**  **(N = 4)** |
| --- | --- | --- | --- | --- |
| **Factor** | **N** | **Summary** | **Summary** | **Summary** |
| Age ^1^ |  |  |  |  |
| Female Gender ^2^ |  |  |  |  |
| Caucasian Race ^2^ |  |  |  |  |
| Years of Education ^1^ |  |  |  |  |
| Years of Cognitive Symptoms ^1^ |  |  |  |  |
| MMSE ^1^ |  |  |  |  |
| ADAS-Cog ^1^ |  |  |  |  |
| CDR-SOB ^1^ |  |  |  |  |
| NPI ^1^ |  |  |  |  |
| ADCS-ADL^1^ |  |  |  |  |
| ApoE ^2^ |  |  |  |  |
| Heterozygotes |  |  |  |  |
| Homozygotes |  |  |  |  |
| Non-carriers |  |  |  |  |

^1^ Mean (SD)

^2^ Percentage

Figure 1. CONSORT Diagram flow chart showing subject participation in the study

10.2. Primary Outcome Results

Table 2. Comparisons of mean changes from baseline to 4 weeks in amyloid burden between treatment groups.

|  | **Bexarotene** | | **Control** | | **Difference** |  |
| --- | --- | --- | --- | --- | --- | --- |
| **Factor** | **N** | **Mean (95% CI)** | **N** | **Mean (95% CI)** | **Mean (95% CI)** | **P-value** |
| All Subjects Composite |  |  |  |  |  |  |

Figure 2. Boxplots showing changes from baseline to 4 weeks in amyloid burden by treatment group.

10.3. Secondary Outcome Results

Table 3. Comparisons of mean changes from baseline to 4 weeks in amyloid burden between treatment groups.

|  | **Bexarotene** | | **Control** | | **Difference** |  |
| --- | --- | --- | --- | --- | --- | --- |
| **Factor** | **N** | **Mean (95% CI)** | **N** | **Mean (95% CI)** | **Mean (95% CI)** | **P-value** |
| All Subjects |  |  |  |  |  |  |
| Frontal Medial Orbital |  |  |  |  |  |  |
| Anterior Cingulate |  |  |  |  |  |  |
| Parietal |  |  |  |  |  |  |
| Posterior Cingulate |  |  |  |  |  |  |
| Precuneus |  |  |  |  |  |  |
| Temporal |  |  |  |  |  |  |
| e4 carriers |  |  |  |  |  |  |
| Composite |  |  |  |  |  |  |
| Frontal Medial Orbital |  |  |  |  |  |  |
| Anterior Cingulate |  |  |  |  |  |  |
| Parietal |  |  |  |  |  |  |
| Posterior Cingulate |  |  |  |  |  |  |
| Precuneus |  |  |  |  |  |  |
| Temporal |  |  |  |  |  |  |
| Heterozygote e4 |  |  |  |  |  |  |
| Composite |  |  |  |  |  |  |
| Frontal Medial Orbital |  |  |  |  |  |  |
| Anterior Cingulate |  |  |  |  |  |  |
| Parietal |  |  |  |  |  |  |
| Posterior Cingulate |  |  |  |  |  |  |
| Precuneus |  |  |  |  |  |  |
| Temporal |  |  |  |  |  |  |
| Homozygote e4 |  |  |  |  |  |  |
| Composite |  |  |  |  |  |  |
| Frontal Medial Orbital |  |  |  |  |  |  |
| Anterior Cingulate |  |  |  |  |  |  |
| Parietal |  |  |  |  |  |  |
| Posterior Cingulate |  |  |  |  |  |  |
| Precuneus |  |  |  |  |  |  |
| Temporal |  |  |  |  |  |  |
| non e4 carriers |  |  |  |  |  |  |
| Composite |  |  |  |  |  |  |
| Frontal Medial Orbital |  |  |  |  |  |  |
| Anterior Cingulate |  |  |  |  |  |  |
| Parietal |  |  |  |  |  |  |
| Posterior Cingulate |  |  |  |  |  |  |
| Precuneus |  |  |  |  |  |  |
| Temporal |  |  |  |  |  |  |
| Number judged positive |  |  |  |  |  |  |

Table 4. Comparisons of changes from baseline to 4 weeks in clinical measures between treatment groups.

|  | **Bexarotene** | | **Control** | | **Difference** |  |
| --- | --- | --- | --- | --- | --- | --- |
| **Factor** | **N** | **Mean (95% CI)** | **N** | **Mean (95% CI)** | **Mean (95% CI)** | **P-value** |
| All Subjects |  |  |  |  |  |  |
| MMSE |  |  |  |  |  |  |
| ADAS-Cog |  |  |  |  |  |  |
| CDR-SOB |  |  |  |  |  |  |
| NPI |  |  |  |  |  |  |
| ADCS-ADL |  |  |  |  |  |  |
| e4 carriers |  |  |  |  |  |  |
| MMSE |  |  |  |  |  |  |
| ADAS-Cog |  |  |  |  |  |  |
| CDR-SOB |  |  |  |  |  |  |
| NPI |  |  |  |  |  |  |
| ADCS-ADL |  |  |  |  |  |  |
| Heterozygote e4 |  |  |  |  |  |  |
| MMSE |  |  |  |  |  |  |
| ADAS-Cog |  |  |  |  |  |  |
| CDR-SOB |  |  |  |  |  |  |
| NPI |  |  |  |  |  |  |
| ADCS-ADL |  |  |  |  |  |  |
| Homozygote e4 |  |  |  |  |  |  |
| MMSE |  |  |  |  |  |  |
| ADAS-Cog |  |  |  |  |  |  |
| CDR-SOB |  |  |  |  |  |  |
| NPI |  |  |  |  |  |  |
| ADCS-ADL |  |  |  |  |  |  |
| non e4 carriers |  |  |  |  |  |  |
| MMSE |  |  |  |  |  |  |
| ADAS-Cog |  |  |  |  |  |  |
| CDR-SOB |  |  |  |  |  |  |
| NPI |  |  |  |  |  |  |
| ADCS-ADL |  |  |  |  |  |  |

Table 5. Evaluations of changes from baseline to 8 weeks in clinical measures in the group receiving Bexarotene 150mg bid at baseline and the open label phase.

|  | **Baseline** | | **8 Weeks** | | **Difference** |  |
| --- | --- | --- | --- | --- | --- | --- |
| **Factor** | **N** | **Mean (95% CI)** | **N** | **Mean (95% CI)** | **Mean (95% CI)** | **P-value** |
| All Subjects |  |  |  |  |  |  |
| MMSE |  |  |  |  |  |  |
| ADAS-Cog |  |  |  |  |  |  |
| CDR-SOB |  |  |  |  |  |  |
| NPI |  |  |  |  |  |  |
| ADCS-ADL |  |  |  |  |  |  |
| e4 carriers |  |  |  |  |  |  |
| MMSE |  |  |  |  |  |  |
| ADAS-Cog |  |  |  |  |  |  |
| CDR-SOB |  |  |  |  |  |  |
| NPI |  |  |  |  |  |  |
| ADCS-ADL |  |  |  |  |  |  |
| Heterozygote e4 |  |  |  |  |  |  |
| MMSE |  |  |  |  |  |  |
| ADAS-Cog |  |  |  |  |  |  |
| CDR-SOB |  |  |  |  |  |  |
| NPI |  |  |  |  |  |  |
| ADCS-ADL |  |  |  |  |  |  |
| Homozygote e4 |  |  |  |  |  |  |
| MMSE |  |  |  |  |  |  |
| ADAS-Cog |  |  |  |  |  |  |
| CDR-SOB |  |  |  |  |  |  |
| NPI |  |  |  |  |  |  |
| ADCS-ADL |  |  |  |  |  |  |
| non e4 carriers |  |  |  |  |  |  |
| MMSE |  |  |  |  |  |  |
| ADAS-Cog |  |  |  |  |  |  |
| CDR-SOB |  |  |  |  |  |  |
| NPI |  |  |  |  |  |  |
| ADCS-ADL |  |  |  |  |  |  |

Table 6. Comparisons of changes from baseline to 4 weeks in amyloid burden between treatment groups (alternative standard).

|  | **Bexarotene** | | **Control** | | **Difference** |  |
| --- | --- | --- | --- | --- | --- | --- |
| **Factor** | **N** | **Mean (95% CI)** | **N** | **Mean (95% CI)** | **Mean (95% CI)** | **P-value** |
| All Subjects |  |  |  |  |  |  |
| Composite |  |  |  |  |  |  |
| Frontal Medial Orbital |  |  |  |  |  |  |
| Anterior Cingulate |  |  |  |  |  |  |
| Parietal |  |  |  |  |  |  |
| Posterior Cingulate |  |  |  |  |  |  |
| Precuneus |  |  |  |  |  |  |
| Temporal |  |  |  |  |  |  |
| e4 carriers |  |  |  |  |  |  |
| Composite |  |  |  |  |  |  |
| Frontal Medial Orbital |  |  |  |  |  |  |
| Anterior Cingulate |  |  |  |  |  |  |
| Parietal |  |  |  |  |  |  |
| Posterior Cingulate |  |  |  |  |  |  |
| Precuneus |  |  |  |  |  |  |
| Temporal |  |  |  |  |  |  |
| Heterozygote e4 |  |  |  |  |  |  |
| Composite |  |  |  |  |  |  |
| Frontal Medial Orbital |  |  |  |  |  |  |
| Anterior Cingulate |  |  |  |  |  |  |
| Parietal |  |  |  |  |  |  |
| Posterior Cingulate |  |  |  |  |  |  |
| Precuneus |  |  |  |  |  |  |
| Temporal |  |  |  |  |  |  |
| Homozygote e4 |  |  |  |  |  |  |
| Composite |  |  |  |  |  |  |
| Frontal Medial Orbital |  |  |  |  |  |  |
| Anterior Cingulate |  |  |  |  |  |  |
| Parietal |  |  |  |  |  |  |
| Posterior Cingulate |  |  |  |  |  |  |
| Precuneus |  |  |  |  |  |  |
| Temporal |  |  |  |  |  |  |
| non e4 carriers |  |  |  |  |  |  |
| Composite |  |  |  |  |  |  |
| Frontal Medial Orbital |  |  |  |  |  |  |
| Anterior Cingulate |  |  |  |  |  |  |
| Parietal |  |  |  |  |  |  |
| Posterior Cingulate |  |  |  |  |  |  |
| Precuneus |  |  |  |  |  |  |
| Temporal |  |  |  |  |  |  |
| Number judged positive |  |  |  |  |  |  |

Table 7. Comparisons of changes from baseline to 4 weeks in serum and plasma biomarkers between treatment groups.

|  | **Bexarotene** | | **Control** | | **Difference** | |  |
| --- | --- | --- | --- | --- | --- | --- | --- |
| **Factor** | **N** | **Mean (95% CI)** | **N** | **Mean (95% CI)** | **N** | **Mean (95% CI)** | **P-value** |
| All Subjects |  |  |  |  |  |  |  |
| Aβ 40 |  |  |  |  |  |  |  |
| Aβ 42 |  |  |  |  |  |  |  |
| sAPP-α |  |  |  |  |  |  |  |
| sAPP-β |  |  |  |  |  |  |  |
| Isoprostanes |  |  |  |  |  |  |  |
| cytokines |  |  |  |  |  |  |  |
| e4 carriers |  |  |  |  |  |  |  |
| Aβ 40 |  |  |  |  |  |  |  |
| Aβ 42 |  |  |  |  |  |  |  |
| sAPP-α |  |  |  |  |  |  |  |
| sAPP-β |  |  |  |  |  |  |  |
| Isoprostanes |  |  |  |  |  |  |  |
| cytokines |  |  |  |  |  |  |  |
| Heterozygote e4 |  |  |  |  |  |  |  |
| Aβ 40 |  |  |  |  |  |  |  |
| Aβ 42 |  |  |  |  |  |  |  |
| sAPP-α |  |  |  |  |  |  |  |
| sAPP-β |  |  |  |  |  |  |  |
| Isoprostanes |  |  |  |  |  |  |  |
| cytokines |  |  |  |  |  |  |  |
| Homozygote e4 |  |  |  |  |  |  |  |
| Aβ 40 |  |  |  |  |  |  |  |
| Aβ 42 |  |  |  |  |  |  |  |
| sAPP-α |  |  |  |  |  |  |  |
| sAPP-β |  |  |  |  |  |  |  |
| Isoprostanes |  |  |  |  |  |  |  |
| cytokines |  |  |  |  |  |  |  |
| non e4 carriers |  |  |  |  |  |  |  |
| Aβ 40 |  |  |  |  |  |  |  |
| Aβ 42 |  |  |  |  |  |  |  |
| sAPP-α |  |  |  |  |  |  |  |
| sAPP-β |  |  |  |  |  |  |  |
| Isoprostanes |  |  |  |  |  |  |  |
| cytokines |  |  |  |  |  |  |  |

Table 8. Evaluations of changes from baseline to 8 weeks in serum and plasma biomarkers in the group receiving Bexarotene 150mg bid at baseline and the open label phase.

|  | **Baseline** | | **8 Weeks** | | **Difference** |  |
| --- | --- | --- | --- | --- | --- | --- |
| **Factor** | **N** | **Mean (95% CI)** | **N** | **Mean (95% CI)** | **Mean (95% CI)** | **P-value** |
| All Subjects |  |  |  |  |  |  |
| Aβ 40 |  |  |  |  |  |  |
| Aβ 42 |  |  |  |  |  |  |
| sAPP-α |  |  |  |  |  |  |
| sAPP-β |  |  |  |  |  |  |
| Isoprostanes |  |  |  |  |  |  |
| cytokines |  |  |  |  |  |  |
| e4 carriers |  |  |  |  |  |  |
| Aβ 40 |  |  |  |  |  |  |
| Aβ 42 |  |  |  |  |  |  |
| sAPP-α |  |  |  |  |  |  |
| sAPP-β |  |  |  |  |  |  |
| Isoprostanes |  |  |  |  |  |  |
| cytokines |  |  |  |  |  |  |
| Heterozygote e4 |  |  |  |  |  |  |
| Aβ 40 |  |  |  |  |  |  |
| Aβ 42 |  |  |  |  |  |  |
| sAPP-α |  |  |  |  |  |  |
| sAPP-β |  |  |  |  |  |  |
| Isoprostanes |  |  |  |  |  |  |
| cytokines |  |  |  |  |  |  |
| Homozygote e4 |  |  |  |  |  |  |
| Aβ 40 |  |  |  |  |  |  |
| Aβ 42 |  |  |  |  |  |  |
| sAPP-α |  |  |  |  |  |  |
| sAPP-β |  |  |  |  |  |  |
| Isoprostanes |  |  |  |  |  |  |
| cytokines |  |  |  |  |  |  |
| non e4 carriers |  |  |  |  |  |  |
| Aβ 40 |  |  |  |  |  |  |
| Aβ 42 |  |  |  |  |  |  |
| sAPP-α |  |  |  |  |  |  |
| sAPP-β |  |  |  |  |  |  |
| Isoprostanes |  |  |  |  |  |  |
| cytokines |  |  |  |  |  |  |

Table 9. Summaries of cholesterol elevation and safety endpoints by group through study completion.

|  | **Bexarotene** | | **Control** | |  |
| --- | --- | --- | --- | --- | --- |
| **Factor** | **N** | **%** | **N** | **%** | **P-value** |
| All Subjects |  |  |  |  |  |
| Cholesterol |  |  |  |  |  |
| 3x+ greater normal |  |  |  |  |  |
| 10x+ greater normal |  |  |  |  |  |
| e4 carriers |  |  |  |  |  |
| Cholesterol |  |  |  |  |  |
| 3x+ greater normal |  |  |  |  |  |
| 10x+ greater normal |  |  |  |  |  |
| non e4 carriers |  |  |  |  |  |
| Cholesterol |  |  |  |  |  |
| 3x+ greater normal |  |  |  |  |  |
| 10x+ greater normal |  |  |  |  |  |

Table 10. Summaries of mean cholesterol levels by group over time are shown.

|  | **Bexarotene** | | **Control** | |
| --- | --- | --- | --- | --- |
| **Factor** | **N** | **Mean (95%CI)** | **N** | **Mean (95%CI)** |
| All Subjects |  |  |  |  |
| Baseline |  |  |  |  |
| Week 2 |  |  |  |  |
| Week 4 |  |  |  |  |
| Week 8 |  |  |  |  |
| Safety Follow-Up |  |  |  |  |
| e4 carriers |  |  |  |  |
| Baseline |  |  |  |  |
| Week 2 |  |  |  |  |
| Week 4 |  |  |  |  |
| Week 8 |  |  |  |  |
| Safety Folllow-Up |  |  |  |  |
| non e4-carriers |  |  |  |  |
| Baseline |  |  |  |  |
| Week 2 |  |  |  |  |
| Week 4 |  |  |  |  |
| Week 8 |  |  |  |  |
| Safety Folllow-Up |  |  |  |  |

Figure 3. Line plot showing cholesterol levels by subject over time are shown.

Table 11. Summaries of safety endpoints by group during the study.

|  | **Bexarotene** | | **Control** | |  |
| --- | --- | --- | --- | --- | --- |
| **Factor** | **N** | **%** | **N** | **%** | **P-value** |
| All Subjects |  |  |  |  |  |
| Adverse Event System |  |  |  |  |  |
| Increase in Triglyceride Levels (> 200 mg/dL) |  |  |  |  |  |
| Increase in Cholesterol Levels (>300 mg/dL) |  |  |  |  |  |
| Delusions |  |  |  |  |  |
| Dizziness |  |  |  |  |  |
| Blister on Toe |  |  |  |  |  |
| Dry Cough |  |  |  |  |  |
| 1+ Adverse Events |  |  |  |  |  |
| 1+ Serious Adverse Events |  |  |  |  |  |
| 1+ Drug Related Adverse Events |  |  |  |  |  |
| 1+ Cholesterol Related Adverse Events |  |  |  |  |  |
| Adverse Event Rate |  |  |  |  |  |
| Serious Adverse Event Rate |  |  |  |  |  |
| Drug Related Adverse Event Rate |  |  |  |  |  |
| Cholesterol Related Adverse Event Rate |  |  |  |  |  |
| e4 carriers |  |  |  |  |  |
| Adverse Event System |  |  |  |  |  |
| Increase in Triglyceride Levels (> 200 mg/dL) |  |  |  |  |  |
| Increase in Cholesterol Levels (>300 mg/dL) |  |  |  |  |  |
| Delusions |  |  |  |  |  |
| Dizziness |  |  |  |  |  |
| Blister on Toe |  |  |  |  |  |
| Dry Cough |  |  |  |  |  |
| 1+ Adverse Events |  |  |  |  |  |
| 1+ Serious Adverse Events |  |  |  |  |  |
| 1+ Drug Related Adverse Events |  |  |  |  |  |
| 1+ Cholesterol Related Adverse Events |  |  |  |  |  |
| Adverse Event Rate |  |  |  |  |  |
| Serious Adverse Event Rate |  |  |  |  |  |
| Drug Related Adverse Event Rate |  |  |  |  |  |
| Cholesterol Related Adverse Event Rate |  |  |  |  |  |
| non e4 carriers |  |  |  |  |  |
| Adverse Event System |  |  |  |  |  |
| Increase in Triglyceride Levels (> 200 mg/dL) |  |  |  |  |  |
| Increase in Cholesterol Levels (>300 mg/dL) |  |  |  |  |  |
| Delusions |  |  |  |  |  |
| Dizziness |  |  |  |  |  |
| Blister on Toe |  |  |  |  |  |
| Dry Cough |  |  |  |  |  |
| 1+ Adverse Events |  |  |  |  |  |
| 1+ Serious Adverse Events |  |  |  |  |  |
| 1+ Drug Related Adverse Events |  |  |  |  |  |
| 1+ Cholesterol Related Adverse Events |  |  |  |  |  |
| Adverse Event Rate |  |  |  |  |  |
| Serious Adverse Event Rate |  |  |  |  |  |
| Drug Related Adverse Event Rate |  |  |  |  |  |
| Cholesterol Related Adverse Event Rate |  |  |  |  |  |

10.4. Planned Exploratory Analyses

Table 12. Spearman correlations between changes from baseline to 4 weeks among clinical measures, cholesterol levels, and amyloid burden.

| **Factor 1** | **Factor 2** | **r** | **95% CI** | **P-value** |
| --- | --- | --- | --- | --- |
| Cholesterol Change | MMSE |  |  |  |
| Cholesterol Change | ADAS-Cog |  |  |  |
| Cholesterol Change | CDR-SOB |  |  |  |
| Cholesterol Change | NPI |  |  |  |
| Cholesterol Change | ADCS-ADL |  |  |  |
| Cholesterol Change | Amyloid Burden |  |  |  |
| Amyloid Burden | MMSE |  |  |  |
| Amyloid Burden | ADAS-Cog |  |  |  |
| Amyloid Burden | CDR-SOB |  |  |  |
| Amyloid Burden | NPI |  |  |  |
| Amyloid Burden | ADCS-ADL |  |  |  |

Figure 4. Scatterplots showing relationships between cholesterol level changes and clinical measure changes. (2x3 panel)

Figure 5. Scatterplots showing relationships between amyloid burden changes and clinical measure changes. (2x3 panel)

Figure 6. Scatterplots showing relationships between amyloid burden changes and cholesterol level changes. (single panel)

10.5. Supplementary Tables

Table S1. Per protocol comparisons of changes from baseline to 4 weeks in amyloid burden measures between treatment groups (see Table 3).

Table S2. Per protocol comparisons of changes from baseline to 4 weeks in amyloid burden measures between treatment groups (alternative standard)

(see Table 6).

Table S3. Per protocol comparisons of changes from baseline to 4 weeks in clinical measures between treatment groups (see Table 4).

Table S4. Per protocol evaluations of changes from baseline to 8 weeks in clinical measures in the group receiving Bexarotene 150mg bid at baseline and the open label phase (see Table 5).

Table S5. Adjusted comparisons of changes from baseline to 4 weeks in amyloid burden measures between treatment groups (see Table 3).

Table S6. Adjusted comparisons of changes from baseline to 4 weeks in amyloid burden measures between treatment groups (alternative standard)

(see Table 6).

Table S7. Adjusted comparisons of changes from baseline to 4 weeks in clinical measures between treatment groups (see Table 4).

Table S8. Adjusted evaluations of changes from baseline to 8 weeks in clinical measures in the the group receiving Bexarotene 150mg bid at baseline and the open label phase (see Table 5).

**Appendix 1. Summary of Statistical Analysis Plan Changes**

01AUG2014

- Updated version to 1.1.
- Document was updated to include primary outcome. This included additional text to the narrative and updated tables.
- Text and tables were updated to explicitly reflect stratified analysis by heterozygote and homozygote e4 status.
- Text was added to evaluate the trajectory of group receiving Bearotene throughout the study to evaluate change over the 8 week time period.

15OCT2014

- Updated version to 1.2.
- Clarified the text language in Section 2.1 to define use of composite white matter as the primary outcome.
- Removed Bex-Bex language from the proposal.
- Added new section 3.4 for comparisons between groups and within group on Bexarotene for the entire study on the change in serum and plasma biomarkers, and added in tables for between and within group change.
- Added new section 5.1 for Populations of Interest and defined intent-to-treat, modified intent-to-treat and per-protocol populations.
- Clarified that efficacy endpoints will be analyzed using the modified intent-to-treat populations and safety endpoints will be evaluated using the intent-to-treat population.
- Added in information regarding CONSORT diagram to text and figures.
- Updated content of tables and split tables reflecting cholesterol elevation and adverse events.
- Renumbered tables as appropriate.

09DEC2014

- Updated version to 1.3.
- Reordered non-carrier rows to end of all tables.
- Added in actual adverse events to Table 11
- Added in mean cholesterol levels by group in Table 10 and line plot as Figure 3, and adjusted other table and figure numbers
- Changed references to global to composite in tables. Added region names to the tables.
- Finalized white matter as primary definition for amyloid burden
